# Synthesis of Tridecacene by Multistep Single-Molecule Manipulation

**DOI:** 10.1021/jacs.3c09392

**Published:** 2024-01-12

**Authors:** Zilin Ruan, Jakob Schramm, John B. Bauer, Tim Naumann, Holger F. Bettinger, Ralf Tonner-Zech, J. Michael Gottfried

**Affiliations:** † Fachbereich Chemie, Philipps-Universität Marburg, Hans-Meerwein-Str. 4, 35032 Marburg, Germany; ‡ Universität Leipzig, Fakultät für Chemie und Mineralogie, Wilhelm-Ostwald-Institut für Physikalische und Theoretische Chemie, Linnéstraße 2, 04103 Leipzig, Germany; § Institut für Organische Chemie, Universität Tübingen, Auf der Morgenstelle 18, 72076 Tübingen, Germany

## Abstract

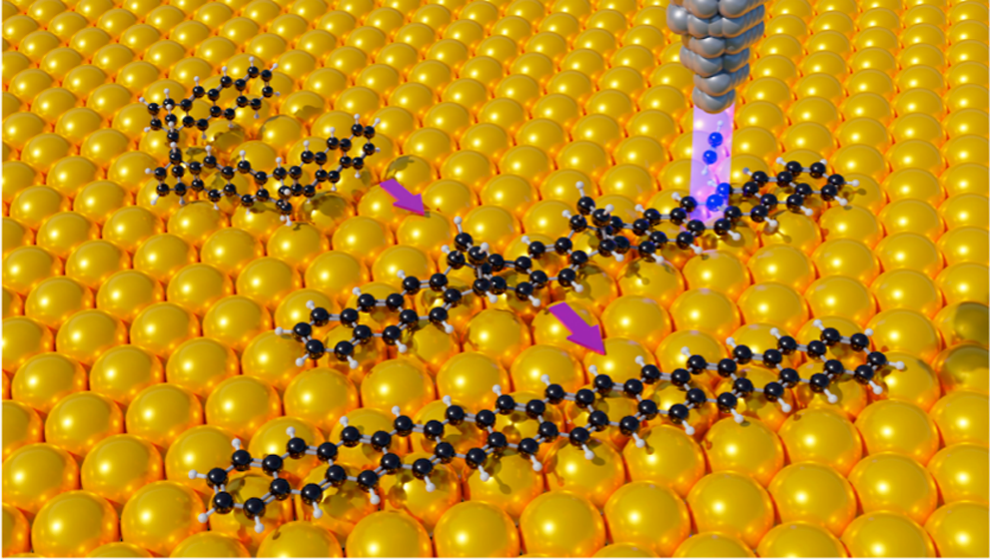

Acenes represent a unique class of polycyclic aromatic hydrocarbons that have fascinated chemists and physicists due to their exceptional potential for use in organic electronics. While recent advances in on-surface synthesis have resulted in higher acenes up to dodecacene, a comprehensive understanding of their fundamental properties necessitates their expansion toward even longer homologues. Here, we demonstrate the on-surface synthesis of tridecacene via atom-manipulation-induced conformational preparation and dissociation of a trietheno-bridged precursor on a Au(111) surface. The generated tridecacene has been investigated by scanning tunneling microscopy and spectroscopy (STM/STS), combined with first-principles calculations. We observe that the STS transport gap (1.09 eV) shrinks again following the gap reopening of dodecacene (1.4 eV). Spin-polarized density functional theory calculations confirm an antiferromagnetic open-shell ground-state electronic configuration for tridecacene in the gas phase. Interestingly, tridecacene’s open-shell character is significantly reduced upon interaction with the Au(111) surface despite being only physisorbed. The interaction with the surface leads to a lowering of the magnetization of tridecacene, a reduced gap between the highest occupied molecular orbital (HOMO) and the lowest unoccupied molecular orbital (LUMO), compared to the gas phase, and a reduced relative energy to the nonmagnetic state, making it nearly isoenergetic. These observations show qualitatively that the influence of the Au(111) substrate on the properties of long acenes is significant, which is important for interpreting the measured STS transport gaps. Our work contributes to a fundamental understanding of the electronic properties of long acenes, confirming a nonmonotonous length-dependent HOMO–LUMO gap, and to the development of multistep tip-assisted synthesis of elusive compounds.

## Introduction

Acenes are an important class of polycyclic aromatic hydrocarbons (PAHs) constituted by linearly fused benzene rings.^1^ They have attracted considerable interest in the fields of organic chemistry and materials science due to their intriguing properties.^2,3^ Their relatively narrow gap between the highest occupied and lowest unoccupied molecular orbitals (HOMO–LUMO gap) among benzenoid PAHs of comparable size makes acenes promising candidates for electronic materials.^4−6^ However, the small size of the HOMO–LUMO gap leads to a deviation from standard electronic concepts,^7^ resulting in a so-called polyradical (also open-shell or antiferromagnetic) character, which is accompanied by high electron correlation.^8−10^ This is in line with the idea that higher acenes can be considered as the narrowest zigzag edge graphene nanoribbons, which are anticipated to possess spin-polarized edge states.^11,12^ Thus, acenes also hold promise for applications in spintronics.^13^ The decrease of the HOMO–LUMO gap and the increase of the (poly)radical character with increasing number of rings do not only lead to challenges for the theoretical description of the electronic structure^2^ but also cause low kinetic stability, which makes experimental characterization of acenes difficult.^14,15^

A recurrent strategy to circumvent the instability of higher acenes has been their functionalization with stabilizing and protecting groups,^16−18^ which has allowed the synthesis of acene derivatives up to nonacene.^19,20^ Significant advances in the preparation of unsubstituted higher acenes up to undecacene have been achieved under matrix isolation conditions.^21−25^ As a powerful complementary approach, higher acenes up to dodecacene have been generated by on-surface chemistry and been investigated with scanning probe microscopy techniques.^26−30^ These methods allow for the precise simultaneous structural and electronic characterization of the generated higher acenes. Previous experimental studies have shown that the scanning tunneling spectroscopy (STS) transport gap, which is associated with the HOMO–LUMO gap, shrinks with increasing length from pentacene (**5ac**, 2.20 eV)^31^ to undecacene (**11ac**, 1.09 eV).^26^ Surprisingly, a reopening of the gap to 1.4 eV, which does not follow the trend of gap shrinking with increasing length, was reported for dodecacene (**12ac**).^27^ The latter finding was explained by an increased contribution of the polyradical character of **12ac**, rather than by the incommensurate energy gap oscillation predicted in other theoretical work.^5,32,33^ A recent study reported the synthesis of polyacene by synthesis inside the channels of a metal–organic framework.^34^ However, the structural and electronic characterization of the resulting mixture of π-stacked acenes with various lengths suffered from an averaging effect due to the characterization techniques used. To further understand the gap evolution and electronic ground state of long acenes, it is thus desirable to selectively generate acenes longer than **12ac** with well-defined lengths and to characterize them with molecular-level precision.

**Scheme 1:**
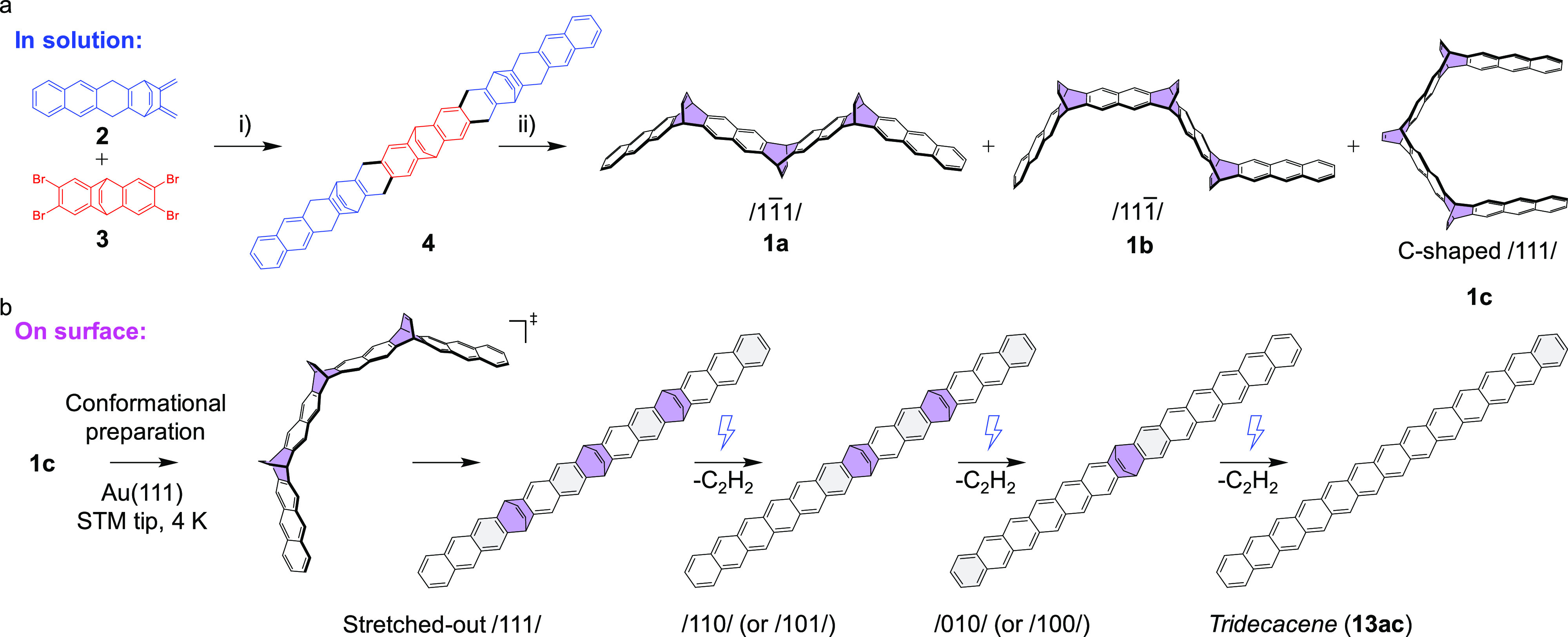
Combined In-Solution and On-Surface Generation of Tridecacene (**13ac**); (a) Mixture of 6,7,8,10,12,13,14,21,22,23,25,27,28,29-Tetradecahydro-7,28:10,25:13,22-triethenotridecacene (**4**) Stereoisomers Were Synthesized from Compounds **2** and **3**; Dehydrogenation of **4** Produces Three Stereoisomers of the Precursor **1**, in which the Etheno-Bridges can be on the Same Side of the Backbone (C-Shaped/111/Isomer **1c**) or on Different Sides (/11®1/and/1®11/Isomers); (i) Toluene, MeLi, 0 °C, 2 h, 21%; (ii) DDQ, CHCl_3_, RT, 2 h, 36%; (b) On-Surface Conformational Preparation of the/111/Isomer **1c** and Subsequent Forced Aromatization by Sequential Removal of Etheno-Bridges Using Manipulation with the STM Tip, which Produces **13ac** via the Dietheno-Bridged (/110/and/101/) and Monoetheno-Bridged (/100/and/010/) Intermediates

In this work, we report the combined in-solution and on-surface generation of tridecacene (**13ac**, Scheme 1), the longest acene generated to date, and the investigation of its structural and electronic properties by scanning tunneling microscopy/spectroscopy (STM/S), noncontact atomic force microscopy (nc-AFM), and spin-polarized density functional theory (DFT) calculations using the PBE functional. The on-surface generation is achieved by rearomatization of a stable hexahydro-trietheno-tridecacene precursor by multistep manipulation in a low-temperature STM. The manipulation sequence includes a three-dimensional conformational transformation step followed by a three-step removal of etheno-bridges via tip-assisted carbon–carbon bond dissociation. The initial manipulation of the molecular conformation is necessary to enable the formation of the desired product. Molecules with one or two etheno-bridges and fewer Clar sextets in their closed-shell resonance structures, compared to the pristine precursor (Scheme 1), occur as intermediates. Our measurements show that the HOMO–LUMO gap shrinks again to 1.09 eV following the gap reopening reported for dodecacene. Spin-polarized DFT calculations confirm an antiferromagnetic open-shell ground-state electron configuration for the gas phase tridecacene, which is significantly reduced upon adsorption on Au(111).

## Results and Discussion

### Edge-On Adsorbed Precursor

**Figure 1:**
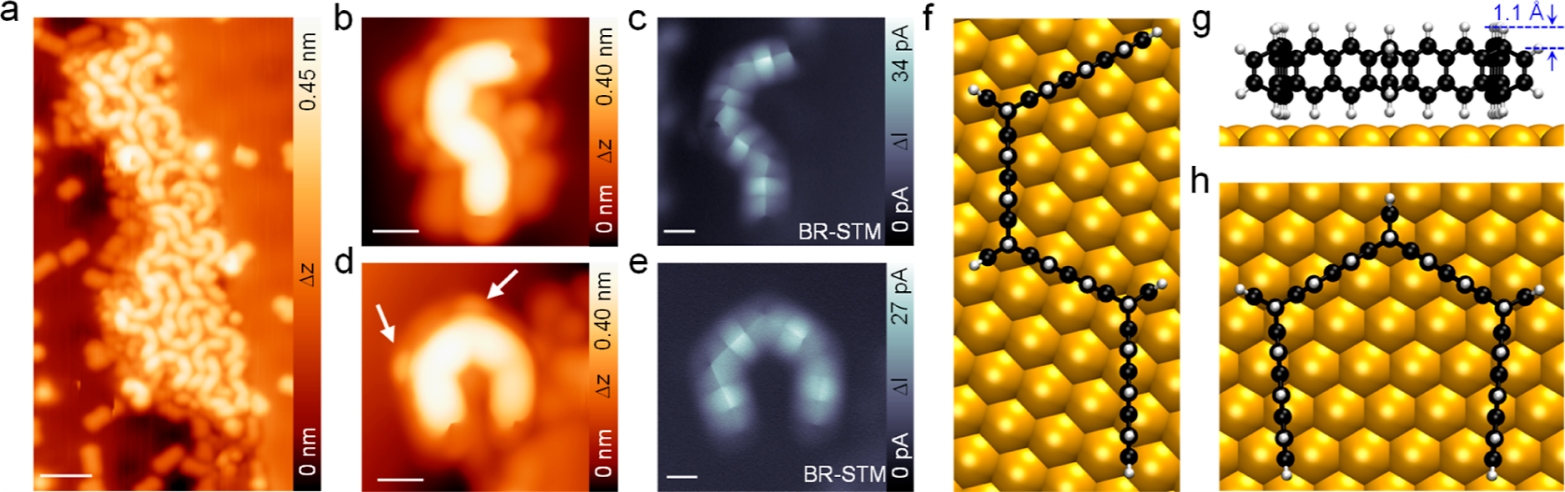
Identification of the precursor after deposition on Au(111). (a) Large-scale STM image of the surface after deposition of the precursor **1**, showing an island with the S-shaped/111®/and the C-shaped/111/stereoisomers. (b,d) Magnified constant-current STM images and (c,e) high-resolution constant-height BR-STM images of the/111®/and/111/isomers. (f, h) Top view on DFT optimized/111®/and/111/isomers on Au(111). (g) Side view of the DFT-optimized/111/isomer on Au(111). The etheno-bridges are highlighted by white arrows in (d). Scale bars: (a) 2 nm; (b,e) 0.7 nm. Scanning parameters: (a) *V*_s_ = 0.15 V, *I*_t_ = 10 pA; (b,d) *V*_s_ = 0.12 V, *I*_t_ = 20 pA.

The trietheno-bridged tridecacene precursor **1** was synthesized in solution (see Methods and Supporting Information). To obtain the presumably highly reactive **13ac**, the stable precursor **1** was vapor-deposited onto the Au(111) surface kept at 140 K. Figure 1a shows a large-scale STM image with a molecular island adjacent to a step edge comprising intact precursor molecules. This island is surrounded by some smaller molecules, which are most likely short acenes formed by partial fragmentation of the precursor at the required high sublimation temperature of 730 K. At a lower coverage, these fragments mainly adsorbed on the terraces, whereas single intact precursor molecules preferentially adsorbed at the step edges (Supporting Information Figure S1). A close-up inspection of the bent molecules in the island and their further isolation by tip manipulation reveals mainly two different stereoisomers of **1**, namely, the/111/isomer (C-shape, **1c**) and the/111®/isomer (S-shape, **1b**), as shown in Figure 1b,c,f,d,e,g,h, respectively, whereas the/11®1/isomer (M-shape, **1a**) was rarely observed (see Supporting Information Figure S2). The observed three different molecular geometries agree well with the expected stereoisomers of **1** seen from the side. It follows that all three isomers adopt an edge-on adsorption geometry, as has been observed previously for the structurally related epoxycyclacenes.^35^ Theoretical calculation with decomposition of the adsorption energy shows that the edge-on adsorbed precursors are purely bound by dispersion forces to the surface with repulsive energy contributions from orbital interactions (see Supporting Information Table S1). To gain more insights into this unusual adsorption configuration, we performed high-resolution bond-resolved STM (BR-STM) imaging with a CO-modified tip.^36^Figure 1c,e shows constant-height BR-STM images of single S-shaped/111®/and C-shaped/111/isomers, respectively. Bond-like features connecting adjacent hydrogen positions are captured. These features can be explained by the flexibility of the CO-terminated tip, which causes the deflection of the CO when the tip reaches the saddle surface produced between adjacent hydrogen atoms^37^ (see also Supporting Information Figure S3). Similar features are captured by the nc-AFM measurements (Supporting Information Figure S4). In the BR-STM images in Figure 1c,e, the hydrogen atoms at the etheno-bridges and the terminal carbon atoms are invisible due to their lower height (∼1.1 Å in DFT, Figure 1g), while they can be identified in the constant-current STM (white arrows, Figure 1d) and constant current nc-AFM images (Supporting Information Figure S4). The symmetric features observed in BR-STM here also demonstrate an edge-on adsorption configuration without tilting of the molecular backbone; i.e., the annulated six-membered rings are perpendicular to the surface, which is in good agreement with the DFT-optimized structural models (Figure 1f,h). We will see below that the edge-on adsorption configuration is metastable for the C-shaped/111/isomer **1c** and may receive additional stabilization from adjacent short acene fragments. Small conformational changes were observed during the further isolation of an edge-on molecule by STM tip manipulation (Supporting Information Figure S5).

### Manipulation of the Precursor Conformation

**Figure 2:**
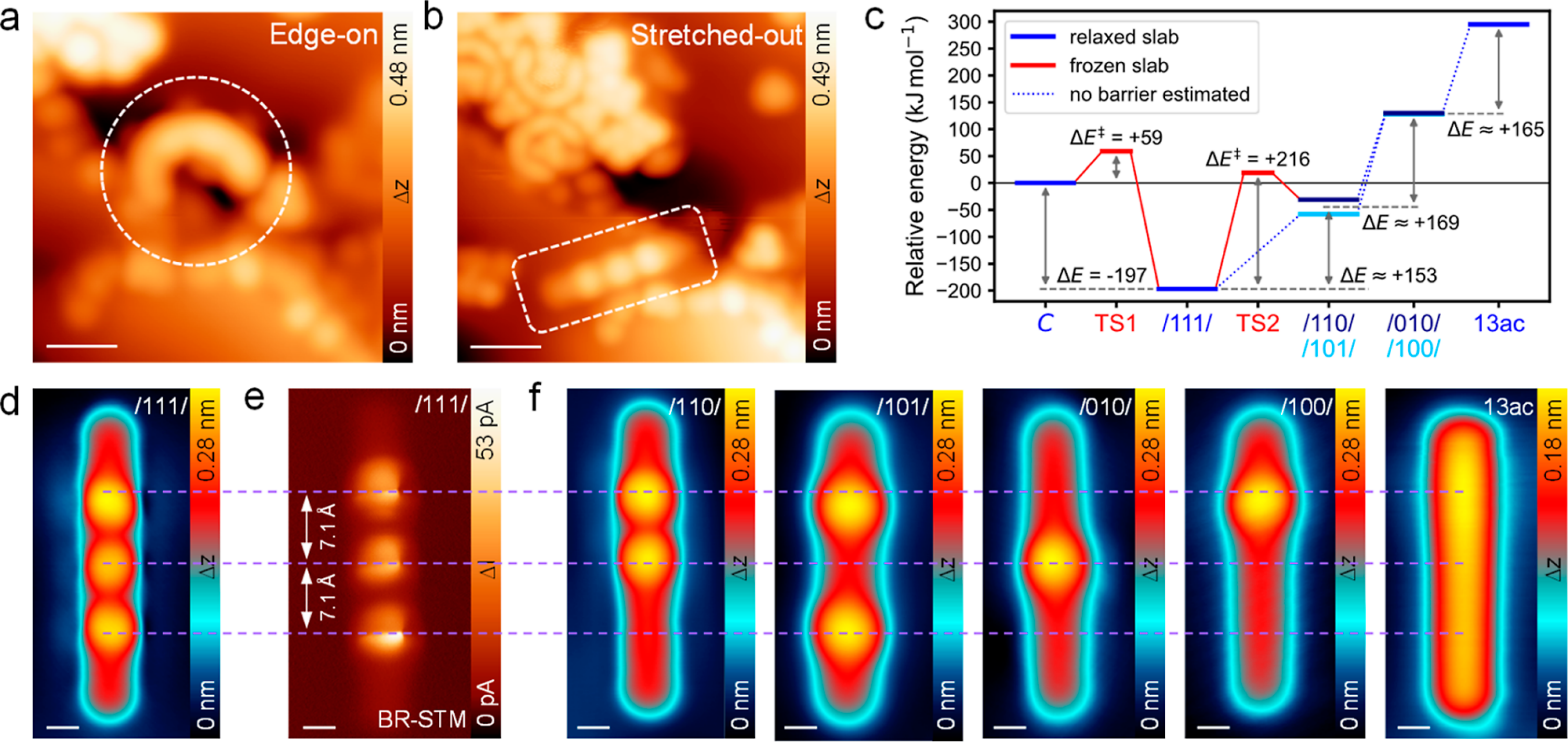
On-surface generation of **13ac** by STM tip manipulation. (a) STM image of an edge-on adsorbed C-shaped/111/isomer and the (b) same molecule stretched out after manipulation. (c) DFT reaction energies Δ*E* of intermediates and reaction barriers Δ*E*^‡^ of transition states (TS) relative to the edge-on C-shaped/111/precursor (C) for the transformation to **13ac** (see also Supporting Information Figures S8, S12 and Tables S2, S3). (d) STM and (e) BR-STM of the stretched out/111/isomer. (f) Series of STM images of products after tip-induced cleavage of the etheno groups; **13ac** is shown on the right. Scale bars: (a) 1.2 nm; (b) 1.5 nm; (d–f) 0.4 nm. Scanning parameters: (a) *V*_s_ = 0.1 V, *I*_t_ = 30 pA; (b) *V*_s_ = 0.05 V, *I*_t_ = 50 pA; (d,f) *V*_s_ = 0.15 V, *I*_t_ = 10 pA; (e) *V*_s_ = 2 mV.

Manipulation with the STM tip close to a step edge was used to induce a conformational transition of the edge-on adsorbed/111/isomer **1c** to its corresponding stretched-out linear conformer, as shown in Scheme 1 and in the STM images in Figure 2a,b (see also Supporting Information Figure S6). The stretched-out conformer is needed for a successful conversion to **13ac** because bond-scission STM manipulation on the edge-on adsorbed conformer resulted in decomposition rather than the desired product. Very rarely, the stretched-out linear conformers can be found on the surface without manipulation (Supporting Information Figure S7), which means that their spontaneous formation is possible but has a low probability at the substrate temperature of 140 K. DFT calculations with the nudged-elastic band (NEB)^38^ approach reveal an energy barrier for the edge-on to stretched-out linear transition of around 60 kJ/mol (Figure 2c, see also Supporting Information Figure S8). This barrier originates from the substantial deformation of the molecule in the transition state (Supporting Information Figure S8) and can also be overcome by thermal activation achieved by annealing at 490 K (Supporting Information Figure S9). High-resolution STM images of the stretched-out/111/isomer reveal three equally spaced etheno-bridges (Figure 2d,e), separated by a distance of ∼7.1 Å, which is in good agreement with the DFT adsorbate structure (∼7.2 Å) and slightly shorter than expected for the distance between the sp^3^ carbon atoms of the gas phase C-shaped/111/structure (∼7.7 Å in DFT) due to the out-of-plane distortion (Supporting Information Figures S10 and S11). The distortion-induced strain is overcompensated by a gain in adsorption dispersion energy, making the transition from the edge-on to stretched-out conformation exothermic with an energy gain of roughly 200 kJ/mol (Figure 2c, see also Supporting Information Table S2). The stretched-out conformation is physisorbed to the surface with a repulsive electronic interaction (Supporting Information Table S2).

### STM-Induced Elimination of the Etheno-Bridges

To obtain the desired **13ac**, we then performed sequential tip manipulations on the stretched-out/111/isomer to remove the etheno groups. Figure 2f shows a series of STM images of all possible intermediate products obtained by STM tip-induced removal of the etheno protecting groups, eventually resulting in the successful generation of **13ac** (see also Supporting Information Figure S10 for the related DFT results). For the cleavage of the three etheno groups, voltage pulses between 2.5 and 3.0 V were applied at the center positions of the etheno groups. DFT calculations show that the removal of an etheno-bridge by release of acetylene is endothermic by around 160 kJ/mol on-surface, nearly independent of which bridge is eliminated (Figure 2c and Supporting Information Table S3). Compared to a hypothetical gas phase reaction,^39^ the reaction energy is decreased because of a gain in adsorption energy (Supporting Information Table S3). This gain in adsorption energy does not result from an increase in dispersion interaction, which one might expect since the molecule becomes flatter, but it is due to release of the deformation-induced strain (Supporting Information Figures S11 and Table S4). All intermediates and the final product **13ac** remain physisorbed to the surface (Supporting Information Table S4). Additionally, it was exemplarily shown that there is an energy barrier for the removal of an etheno-bridge, adding further ∼60 kJ/mol to overcome (Figure 2c and Supporting Information Figure S12). Those results support the observations made when annealing the Au(111) sample with the adsorbed precursor to 490 K: the annealing induced the edge-on to stretched-out transition (barrier ∼60 kJ/mol), but no elimination of etheno-bridges (barrier ∼220 kJ/mol) occurred (Supporting Information Figure S9). Furthermore, due to the chemical robustness of the etheno groups, side reactions were occasionally observed (Supporting Information Figures S13 and S14), which is different from previously reported epoxyacene,^27,29^ hydroacene,^26,30^ or α-diketone precursors,^28^ for which a forced aromatization can be realized with less stress to the annulated backbone. However, the high stability of the etheno-bridges is also advantageous because it reduces decomposition of the precursor during vapor deposition.

**Figure 3:**
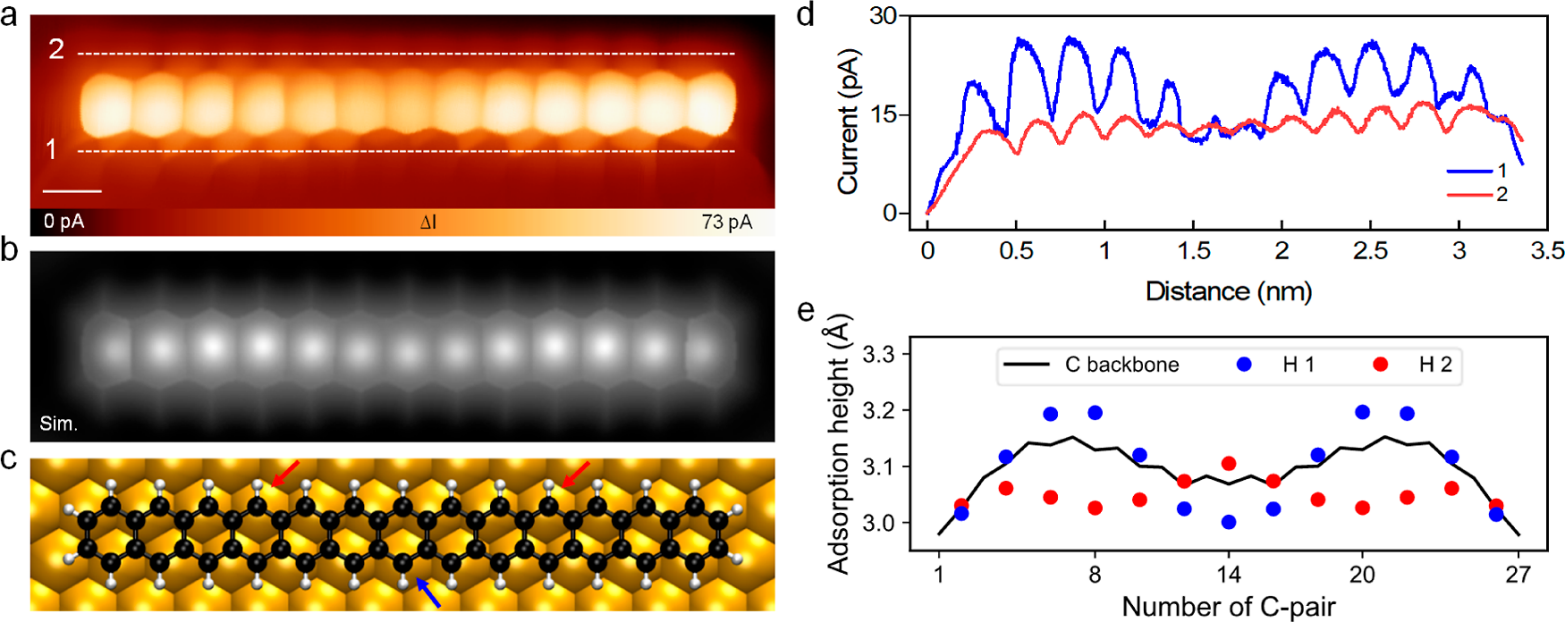
Structure of **13ac** on Au(111). (a) BR-STM image of **13ac** on Au(111). (b) Simulated image of **13ac** on Au(111). (c) Structural model of DFT-optimized **13ac** on Au(111). Blue and red arrows indicate on-top positions of the corresponding carbon and hydrogen atoms at the respective sides of the molecule. (d) Height profiles measured along the dashed lines 1 and 2 in (a). (e) Height-profile obtained from the DFT-optimized structure of the carbon backbone (black). The blue circles are the vertical positions of the H atoms on the long edge of the molecule corresponding to the line profile 1 in (d) (blue), and the red circles are the vertical positions of the H atoms on the other long edge, corresponding to line profile 2 in (d) (red). Scale bars: (a,b) 0.3 nm.

To ultimately prove the successful generation of **13ac** from the/111/precursor, we performed high-resolution STM imaging with a CO-functionalized tip^36,40^ on the molecule shown in Figure 2f (right). Figure 3a presents the corresponding BR-STM image of a single **13ac** molecule in which the 13 lobes corresponding to the 13 benzene rings are clearly resolved. The simulated image^37^ shown in Figure 3b agrees well with the experimental result. It is also found that the central benzene ring appears slightly darker than the other rings, an effect that we attribute to the adsorption on the Au(111) surface. Additionally, the BR-STM image shows an apparent rounding of the terminal benzene rings, an effect that is attributed to the deflection of the CO tip.^37^ A quantitative comparison of the adsorption heights between experiment and theory is difficult because the high-resolution image obtained by a CO-tip BR-STM measurement suffers from the bond amplification at the peripheries as well as from contributions from electronic states, which do not necessarily reflect the actual adsorption structure. In fact, the DFT-optimized structure demonstrates that **13ac** on Au(111) is bent toward the surface in the center as well as at both ends (Figure 3e), while in the BR-STM image (Figure 3a), the two terminal benzene rings appear brightest. The fact that BR-STM fails to reflect the actual corrugation of the DFT structure can be explained by contributions from the electronic states in the experiment. Much better agreement between experiment and theory is obtained when the height-profiles are measured along the edges of **13ac**, approximately at the hydrogen positions (dotted lines in Figure 3a). The resulting experimental height profiles (Figure 3d) match well with the adsorption heights of the H atoms in the DFT-optimized structure (Figure 3e). The height difference between the two long edges is possibly caused by incommensurability of the surface and adsorbate. As a result, on one long edge of the molecule, two C–H groups occupy on-top positions, while on the opposite edge, it is only one C–H group (red and blue arrows in Figure 3c). On-top adsorption leads to stronger dispersion interactions and thus shorter adsorption height. The remaining atoms then show larger adsorption heights, as shown in Figure 3e. Therefore, we attribute this bending of **13ac** only to anisotropy in dispersion attraction and not to the molecule’s radical character discussed below. This is in line with the decomposition of the adsorption energy, which shows dispersion-dominated physisorption with a repulsive electronic interaction (Supporting Information Table S4).

Remarkably, the spontaneous formation of a tetrahydrogenated **13ac** with two incorporated Au atoms (**2Au-4H-13ac**) was observed on the surface (Supporting Information Figures S15–S17). We attribute the formation of these species to the high deposition temperature, which apparently can result in a thermally induced removal of all three etheno-bridges, while the presumably highly reactive **13ac** was further stabilized by the easily accessible hydrogen atoms during preparation and Au atoms from the substrate. By a step-by-step removal of the additional hydrogen atoms and Au atoms, **13ac** can be also generated (Supporting Information Figure S16). A complex of **13ac** with Au atoms can be also generated from a precursor molecule attached to a step edge (Supporting Information Figure S9); however, the strong interaction with the step edge makes further isolation and characterization challenging. The formation of the Au complex illustrates the extremely reactive radical character of **13ac**.^28^

### Electronic Properties

**Figure 4:**
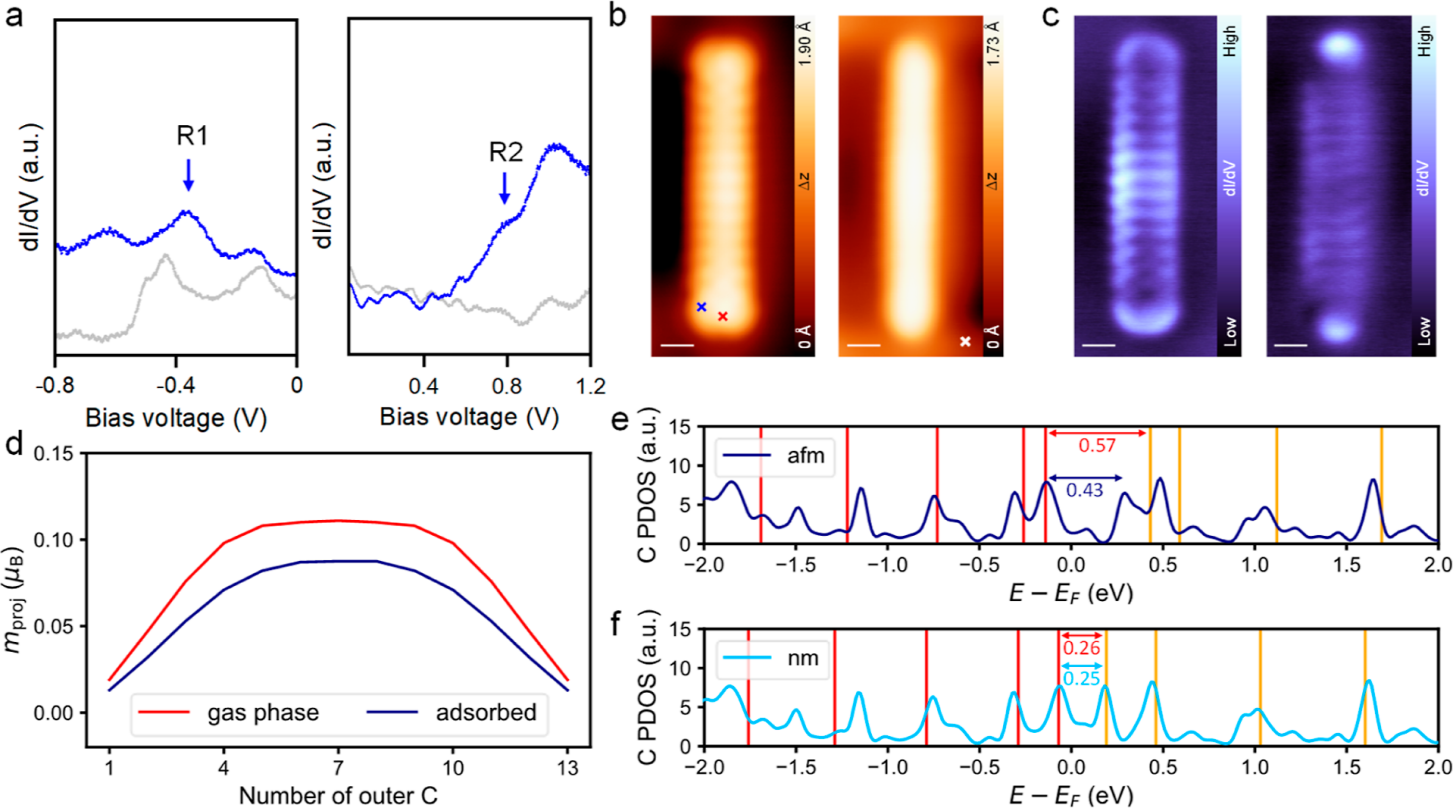
Electronic properties of **13ac**. (a) d*I*/d*V* curves of **13ac** (blue) at negative (left) and positive (right) bias, obtained at the positions marked by blue and red crosses in (b), respectively. The gray curves obtained at the position marked by the white cross in (b) on the bare Au(111) surface are shown for reference. The HOMO (R1) and LUMO (R2) related resonance peaks are marked by blue arrows. (b) STM images of **13ac** at the bias voltages near the resonance peaks R1 (left) and R2 (right) in (a). (c) Constant-current d*I*/d*V* maps recorded at energies close to the resonances shown in (a). (d) Atom-projected magnetization of the outer major-spin carbon atoms for **13ac** in the gas phase (red) and adsorbed on Au(111) (dark blue) **13ac**. (e) Carbon PDOS of the antiferromagnetic state for the adsorbed **13ac** (dark blue) with marking of the occupied (red) and virtual (orange) orbital energies of the gas phase antiferromagnetic (afm) state. (f) Same as in (e) for the nonmagnetic (nm) state (light blue). The gas phase orbital energies were aligned so that the HOMOs had the same energy as the peak of the occupied states closest to the Fermi energy. Therefore, it is artificial in (e) that the LUMO and LUMO + 1 energies of the PDOS are decreased compared to those of the gas phase. Scale bars: (b,c) 0.5 nm. Scanning parameters: (b) *V*_s_ = −0.6 V, *I*_t_ = 100 pA (left) and *V*_s_ = 0.5 V, *I*_t_ = 100 pA (right); (c) *V*_s_ = −0.5 V, *I*_t_ = 120 pA (left) and *V*_s_ = 0.7 V, *I*_t_ = 120 pA (right). (a,c) *V*_rms_ = 20 mV.

The electronic properties of **13ac** were probed by STS measurements. Figure 4a shows a series of d*I*/d*V* curves obtained at the positions marked by crosses in Figure 4b. We observe two tunnel electronic resonance peaks at ∼−0.34 and ∼0.75 eV, which we assign to the HOMO and LUMO, respectively (see Supporting Information Figure S18 for more STS results for gap determination). The resulting STS transport gap of ∼1.09 eV is noticeably smaller than that of **12ac** (1.4 eV),^27^ but it is almost identical to that of **11ac** (1.09 eV).^26^ In a recent preprint, **13ac** was generated by annealing of a hydroacene precursor on Au(111) and studied with STS. In these measurements, the resonance at 0.75 eV was not visible, and the resonance at 1.0 eV was assumed to correspond to the LUMO, leading to the assumption of a larger gap.^41^ In addition, this preprint also reports a spin-excitation feature, which demonstrates an afm ground state of **13ac** on Au(111),^41^ but this feature does not appear in our data. This discrepancy with our work may be rationalized as follows: The extended high-temperature annealing used in the preprint should lead to extensive diffusion and the resulting adsorption on a lowest-energy site (possibly a defect site). In contrast, for the **13ac** generated by local tip-manipulation conducted at 4.2 K, the thermally activated diffusion is absent, thus likely resulting in a different adsorption site. The different adsorption sites, combined with the strong influence of the Au(111) surface on the electronic properties of **13ac** (which will be discussed below), may be the reason for the different measured transport gaps. In line with this, the switch between open- and closed-shell states of a single molecule has recently been observed by changing its adsorption site on NaCl. This change in the adsorption site induced sufficient change in the geometry and the corresponding modification of the ground state electronic configuration.^42^

The unexpected length-dependent opening and closing of the experimental STS transport gap require further discussion. Based on gas-phase closed-shell DFT calculations, it has been proposed that a length-dependent incommensurate oscillation of the energy gap may occur.^5,32^ This oscillation should be accompanied by a change of symmetry of the HOMO and LUMO close to **10ac**.^5^ However, the experimental d*I*/d*V* maps (cf. Figure 4c) do not show this change up to **13ac**. Furthermore, using more accurate theoretical approaches, which include electron correlation effects explicitly, the gap oscillation could be eliminated.^43,44^ However, this apparent lack of description of electron correlation in the closed-shell DFT calculations was critically discussed by the authors.^5,32,33^ They argued that by adsorption of acenes on a substrate, as is the case for the STS gap measurements, the surface screens the electron–electron interaction, weakening electron correlation effects. Additionally, it is known that physisorption of molecules on a metal surface can lead to a reduction of the HOMO–LUMO gap.^45^ This effect of the substrate on the energy gap and radical character of higher acenes has not yet been comprehensively studied. Although van Setten et al.^32^ studied adsorption on Au(111) up to **7ac**, they used only closed-shell DFT computations, which would not be appropriate for longer acenes due to their increasing radical character. The effect of the metal substrate on longer acenes was only modeled for the HOMO–LUMO gap of **11ac** using a classical correction^46^ and on the singlet–triplet gap of **13ac** using a model Hamiltonian approach.^41^ However, both methods lack an ab initio character and an indication of the influence on the ground state radical character. Instead, the spin-polarized variant of DFT could be used, as Jiang and Dai^47^ have shown for the gas phase acenes. In this way, it is possible to evaluate the effect of the Au(111) substrate on the HOMO–LUMO gap as well as the radical character of a higher acene.

Employing spin-polarized DFT, we were able to confirm the results by Jiang and Dai^47^ and find an antiferromagnetic (afm, radical or open-shell) self-consistent field (SCF) solution for **13ac** that is 9 kJ/mol lower than the nonmagnetic (nm, closed-shell) solution for the wave function (Supporting Information Figure S19a). The multiconfigurational character of long chain acenes, which is a challenge for the description by DFT methods, is an intensely investigated question in the literature.^9,14,24,48,49^ A considerable multiradical character has been found, e.g., in DFT/MRCI studies of undecacene.^24,49^ However, the use of broken-symmetry DFT methods delivers qualitatively correct results in many cases, and multiconfigurational approaches are not yet possible for ab initio treatments of molecules on surfaces. This is underlined by the qualitative agreement of our results with a multiconfigurational treatment of **13ac**, where the surface is treated with a model Hamiltonian approach.^41^ Furthermore, by using the atom-projected magnetization, we can qualitatively confirm the results of Trinquier et al.^50^ that a tetraradical character is present for acenes around **14ac** in the gas phase (Supporting Information Figure S19c).

This treatment also eliminates the oscillatory behavior of the HOMO–LUMO gap (Supporting Information Figure S19b). Note that the HOMO–LUMO gap is still underestimated in comparison to STS transport gap measurements, which is a known problem of density functionals using the generalized gradient approximation.^51,52^ However, it is the only class of functionals that makes surface calculations with such a large number of atoms feasible. Therefore, we will not draw conclusions based on a comparison between theoretical and experimental results but rather discuss qualitatively the trends of the theoretical results.

When **13ac** is adsorbed on the Au(111) surface, also an afm state can be found. However, the afm ground state of the adsorbed molecule is only 2 kJ/mol more stable than the nm state, making these states nearly isoenergetic. The reason for the lowering of the energy difference between these states is that although in both cases, the adsorption energy is dominated by the dispersion interaction, there is a larger electronic repulsion between the surface and the molecule for the afm state (Supporting Information Table S5). Furthermore, a significant reduction of the afm (open-shell) character is observed in the atom-projected magnetization (a decrease of ∼26%, Figure 4d). This reduction of the afm character also substantially reduces the HOMO–LUMO gap of the adsorbed **13ac** from 0.57 to 0.43 eV (decrease by ∼25%, Figure 4e). On the other hand, no significant change of orbital energies for the HOMO – 1, HOMO, LUMO, or LUMO + 1 are observed for the adsorbed **13ac** in the nm state (Figure 4f). Nonetheless, these results should only be viewed qualitatively since spin-polarized DFT, as every approximation, has limited accuracy (see Supporting Information Figures S20, S21, and Table S6 for more Results and Discussion). Still, the three observations mentioned above show qualitatively that the influence of the Au(111) substrate is substantial.

It turns out that the situation in an STS transport gap measurement is much more complicated. On the one hand, the reasons discussed by Eisenhut et al.^27^ were that higher states, affected by the polyradical character of the acene, play an important role in the tunneling process. On the other hand, the surface seems to have an influence on the polyradical character of the acene, leading in the case of **13ac** to a decrease of the HOMO–LUMO gap, which should also influence the measured STS transport gap. Therefore, further experimental studies with alternative methods to eliminate the influence of the metal surface as well as theoretical investigations are necessary to understand the measured STS transport gap in detail.

## Conclusions

In conclusion, we have demonstrated the on-surface synthesis of tridecacene (**13ac**) on the Au(111) surface achieved by three-step removal of etheno groups from a trietheno-bridged precursor molecule. The precursor initially adsorbs in a peculiar metastable edge-on adsorption geometry, which can be transformed to the stretched-out linear geometry using STM tip manipulation. This stretched-out conformation is suitable for further STM manipulation, resulting in the removal of the etheno-bridges and formation of **13ac**, which was structurally characterized by STM. STS of **13ac** reveals a transport gap of 1.09 eV, showing a shrinking of the gap compared to that of dodecacene, for which a gap reopening relative to that of undecacene was reported. Spin-polarized DFT calculations confirm an antiferromagnetic open-shell ground-state electron configuration for the gas phase tridecacene. The open-shell character is significantly reduced upon interaction with the Au(111) surface, although **13ac** is physisorbed. The interaction with the Au substrate leads to a lowering of the magnetization (open-shell character) of **13ac**, a reduced HOMO–LUMO gap of the adsorbed molecule compared to the gas phase, and a reduced energy relative to the nonmagnetic state, making them nearly isoenergetic. These three observations show qualitatively that the influence of the metal substrate is significant and could be important in interpreting the behavior of measured STS transport gaps as a prerequisite for possible applications of long acenes.

## Experimental and Computational Details/Methods

### Synthesis of the Precursor

Compounds **2** and **3** were synthesized according to the literature.^53,54^ The synthetic route toward the **13ac** precursor **1** starts from diene **2** and aryne precursor **3** (Scheme 1a). The Diels–Alder reaction between **2** and the aryne generated from **3** with MeLi led to the formation of **4** in a yield of 21%. Compound **4** was dehydrogenated with DDQ to give **13ac** precursor **1** in a yield of 36% as a mixture of three different stereoisomers (Scheme 1a). See the Supporting Information for further details.

### On-Surface Synthesis

#### Sample Preparation

The atomically clean Au(111) (MaTecK) surface was obtained by cycles of argon-ion sputtering and annealing at 800 K. The trietheno-bridged precursor was vapor-deposited from a standard Knudsen cell heated to 730 K, while the Au(111) substrates were held at 140 K. Carbon monoxide (CO) molecules were dosed onto the sample surfaces for tip modification.

#### Scanning Probe Microscopy Characterization

Experiments were performed by using an ultrahigh vacuum low-temperature scanning tunneling microscope (UHV LT-STM, Scienta Omicron) with a base pressure better than 1 × 10^–10^ mbar. All the STM images were acquired with the constant current or constant height mode by using an electrochemically etched tungsten tip at 4.2 K, unless otherwise specified. All given voltages were applied to the sample with respect to the tip. Nanotec Electronica WSxM software^55^ was used to process the images. Constant current mode nc-AFM measurements were performed at 4.2 K with tungsten tips placed on a qPlus tuning fork sensor^56^ driven at its resonance frequency (28,780 Hz) with a constant amplitude of ∼70 pm. The tips were functionalized with a single CO molecule at the tip apex picked up from the Au surface after dosing with CO. The Δ*z* was positive (negative) when the tip–surface distance was increased (decreased) with respect to the STM set point at which the feedback loop was opened. The d*I*/d*V* spectra were recorded by using a lock-in amplifier with a modulation frequency of 579 Hz and an amplitude of 20 mV. d*I*/d*V* maps were collected in constant-current mode.

### Computational Investigations

All unpolarized (nonmagnetic) and spin-polarized (antiferromagnetic) DFT calculations were performed with the Vienna Ab Initio Simulation Package (VASP) Version 5.4.4^57−60^ employing the PBE functional^61,62^ together with the third-generation van der Waals dispersion correction by Grimme (DFT-D3) with Becke–Johnson damping.^63,64^ Plane-wave basis sets within the projector-augmented wave ansatz^65,66^ with an energy cutoff of 400 eV were used. The bulk lattice parameter for Au was optimized with the Birch–Murnaghan approach, yielding 4.101 Å, which is in good agreement to experimental values (4.065 Å at 5 K).^67,68^ The Au(111) surface was modeled using a 4-layered 13 × 13 slab. In this way, all adsorption configurations could be calculated on the same slab to avoid artificial differences. During optimizations, the bottom two layers of the slab were kept frozen in their bulk position. The plane-wave cutoff energy, *k*-grid, vacuum layer thickness, and smearing parameters were determined by convergence studies. These showed that for this slab size modeling, the Brillouin zone at the Γ-point is sufficient. Furthermore, a vacuum layer of 20 Å and the second-order Methfessel–Paxton smearing method with a smearing width of 0.01 eV were employed to improve SCF convergence. The SCF convergence criterion was set to 10^–5^ eV, and all structures were optimized until changes in forces were below 10^–2^ eV/Å. An extensive search for the best adsorption site for all adsorbed molecules was carried out: First, since a dispersion-dominated bond was expected, only the DFT-D3(BJ) energies were calculated for the molecules in on-top, bridge, and hollow positions with incremental 1° rotations up to 360°. Subsequently, a preoptimization of the 7 to 9 lowest energy structures per molecule was done using lower computational settings (force convergence criterion of 5 × 10^–2^ eV/Å and smearing width of 0.2 eV) and a frozen slab. Finally, the 4 to 5 lowest energy structures per molecule were optimized using the settings mentioned above.

Reaction barriers were estimated using the NEB method implemented in the TST Tools extension of VASP.^38^ For each NEB run, 7 images and the default spring constant of 5 eV/Å^2^ were used. However, to save computational costs, no climbing image was used and the atoms of the entire slab were kept frozen at their isolated optimized positions (frozen slab approximation).

The experimentally measured BR-STM image was approximated by a nc-AFM simulation. All nc-AFM simulations were done using the probe particle model^37,69^ and the Hartree potential obtained by VASP. In this model, there are several free parameters. They were determined by using different combinations of typical values and comparing the results to experimental data, resulting in an effective tip charge of −0.1 e and a tip stiffness of 0.5 N/m.

Calculations of **7ac** to **15ac** in the gas phase were done using a box of 64 Å × 35 Å × 30 Å to ensure no interactions between the images.

The spin-polarized DFT calculations must be initialized. This was done by using the optimized nonmagnetic structures and initializing spin up (α) at the outer carbon atoms (those with C–H bond) on one side of the molecule, while initializing spin down (β) on the outer carbon atoms on the other side of the molecule.

The projected density of states (PDOS) and projected magnetization were directly obtained from VASP. However, the *k*-grid was improved to 2 × 2 × 1 since for Γ-only, artificial splitting of some orbitals was observed.

STM simulations were performed using the Tersoff–Hamann approximation^70^ and the partial charge density of the respective bands obtained by VASP. For gas phase molecules, the internal index of the bands was used to obtain the partial charge density, while for the adsorbed molecules, the energy (±0.02 eV) of the orbital peaks in the carbon PDOS was used.

Computational data underlying this manuscript are freely accessible at the NOMAD repository under 10.17172/NOMAD/2023.08.31-2.

## Supporting Information Available

Synthetic procedures and spectroscopic data for all new compounds, including images of NMR spectra, additional SPM data, and additional computational results (PDF)
