# Supplementary material for: Synthesis of Tridecacene by Multistep Single-Molecule Manipulation
Source: J Am Chem Soc. 2024 Jan 12;146(6):3700–9. doi: 10.1021/jacs.3c09392 (PMC10870776; doi:10.1021/jacs.3c09392)
Supplement: Supplementary file 1 — ja3c09392_si_001.pdf [file ja3c09392_si_001.pdf]

Supporting information

Synthesis of Tridecacene by Multistep Single-Molecule Manipulation

Zilin Ruan[1,†], Jakob Schramm[2,†], John B. Bauer[3†], Tim Naumann[1], Holger F. Bettinger[3], Ralf Tonner-Zech[2]*, J. Michael Gottfried[1]**

[1]Philipps-Universität Marburg, Fachbereich Chemie, Hans-Meerwein-Str. 4, 35032 Marburg, Germany, michael.gottfried@chemie.uni-marburg.de

[2]Universität Leipzig, Fakultät für Chemie und Mineralogie, Wilhelm-Ostwald-Institut für Physikalische und Theoretische Chemie, Linnéstraße 2, 04103 Leipzig, Germany, ralf.tonner@uni-leipzig.de

[3]Universität Tübingen, Institut für Organische Chemie, Auf der Morgenstelle 18, 72076 Tübingen, Germany, holger.bettinger@uni-tuebingen.de

1. Synthetic procedures

General. Unless otherwise stated, all chemicals were either used as received from their respective commercial suppliers or purified according to literature recommendation[46]. DCM and toluene were dried using a Braun SPS-800 solvent drying system. Dry $CHCl_3$ was purchased from Acros. Solvents for flash column and thin layer chromatography, including dichloromethane and *n*-hexane were all of HPLC grade quality. Thin layer chromatography was performed on a fluorescence indicator marked precoated silica gel 60 plates and visualized by UV light (254 nm/366 nm). Column chromatography was performed on silica gel (0.040 – 0.063 mm).

NMR spectroscopy. ^1H and ^{13}C NMR spectra were recorded on a Bruker Avance III HDX 600 or on a BRUKER Avance III HDX 700 instrument. Chemical shifts for ^1H NMR are reported as δ relative to tetramethyl silane and calibrated against the signal of $CHCl_3$ at 7.26 ppm. Chemical shifts for ^{13}C NMR are reported as δ relative to tetramethyl silane and calibrated against the signal of $CHCl_3$ at 77.16 ppm. The following abbreviations were used to describe splitting patterns: s = singlet, m = multiplet.

Mass spectrometry. High-resolution APCI spectra were obtained on a maxis 4G from the company Bruker with a TOF mass analyzer.

Synthesis of 6,7,8,10,12,13,14,21,22,23,25,27,28,29-tetradecahydro-7,28:10,25:13,22-triethenotridecacene (4)

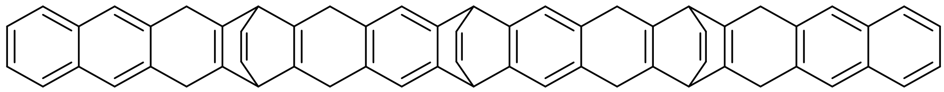

Under argon, 323 mg (1.14 mmol) **2** and 237 mg (456 µmol) of tetrabromide **3** were dissolved in 80 ml of dry toluene. The mixture was cooled to 0 °C and over a period of 20 minutes 1.6 ml (2.56 mmol, 1.6 M in Et_2O) of MeLi was added via syringe. After complete addition, the mixture was stirred at 0 °C for 2 h and then slowly warmed to RT overnight. The reaction mixture was then washed with water and the aqueous phase was separated. The organic phase is removed in vacuo and the crude product was purified by column chromatography (silica gel,

DCM:*n*-hexane first 1:5, later 1:3, then DCM). After removal of the solvent, 73 mg (96 µmol, 21 %) of **4** was obtained as a colorless solid.

The ^{1}H NMR showed slight contaminations, but the purity of the compound was sufficient for the next reaction step. The compound could not be ionized by electron spray or atmospheric pressure chemical ionization.

^{1}H NMR (700 MHz, $CDCl_3$) δ [ppm]: 7.69 (4 H, m), 7.57 (4 H, s), 7.34 (4 H, m), 6.98 (2 H, s), 6.96 (2 H, s), 6.88 (2 H, m), 6.83 (2 H, m), 6.77 (2 H, m), 4.94 (2 H, m), 4.28 (2 H, m), 4.25 (2 H, m), 3.73 (8 H, s), 3.48 (8H, m).

^{13}C NMR (176 MHz, $CDCl_3$) δ [ppm]: 144.1, 144.0, 140.6, 140.5, 140.4, 140.4, 139.5, 139.5, 139.4, 139.4, 133.5, 132.3, 130.2, 130.1, 127.2, 127.1, 126.8, 126.8, 125.1, 125.1, 123.3, 123.2, 54.5, 54.4, 50.3, 33.4, 33.3, 33.3.

Synthesis of 7,10,13,22,25,28-hexahydro-7,28:10,25:13,22-triethenotridecacene (1)

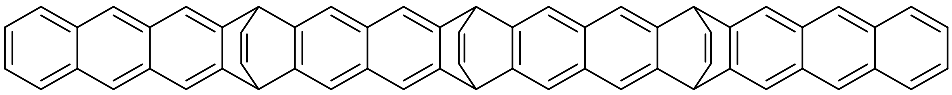

140 mg (180 µmol) of **1** and 540 mg (2.38 mmol) of DDQ were added to 80 ml of dry $CHCl_3$ and were stirred for 2 h at RT. To the reaction mixture, 20 ml of a saturated $NaHCO_3$ solution was added and the mixture was stirred for 10 min at RT. The organic phase was separated, and the aqueous phase was extracted twice, each time with 30 ml $CHCl_3$. The combined organic phases were washed twice with 30 ml of water. The solvent was removed in vacuo and the crude product was purified by column chromatography (silica gel, DCM:*n*-hexane first 1:3, later 1:2). After removal of the solvent, 50 mg (66 µmol, 36 %) of **1** was obtained as a colorless solid.

^{1}H NMR (600 MHz, $CDCl_3$) δ [ppm]: 8.21 (2H, s), 8.21 (2H, s), 7.91 (4H ,m), 7.76 (2H, s), 7.75 (2H, s), 7.59 (2H, s), 7.55 (2H, s), 7.55 (2H, s), 7.51 (2H, s), 7.37 (4H, m), 6.99 (2H, m), 6.91 (4H, m), 5.24 (2H, m), 5.19 (2H, m), 5.17 (2H, m).

^{13}C NMR (150 MHz, $CDCl_3$) δ [ppm]: 141.5, 141.4, 140.7, 140.6, 140.6, 140.6, 140.5, 137.6,

137.5, 137.1, 136.9, 130.7, 130.7, 129.6, 129.5, 128.9, 128.9, 127.1, 127.0, 124.6, 124.6, 124.0, 123.9, 120.2, 120.2, 120.0, 120.0, 119.7, 119.7, 49.3, 49.1, 49.1.

R

$\boldsymbol{R_f}$ **value** (silica gel, DCM:*n*-hexane 1:1): 0.45.

HRMS (APCI) *m*/*z*: $[M+H]^+$ calculated for $C_{60}H_{37}^+$ 757.28898; measured 757.28959.

2. NMR and mass spectra

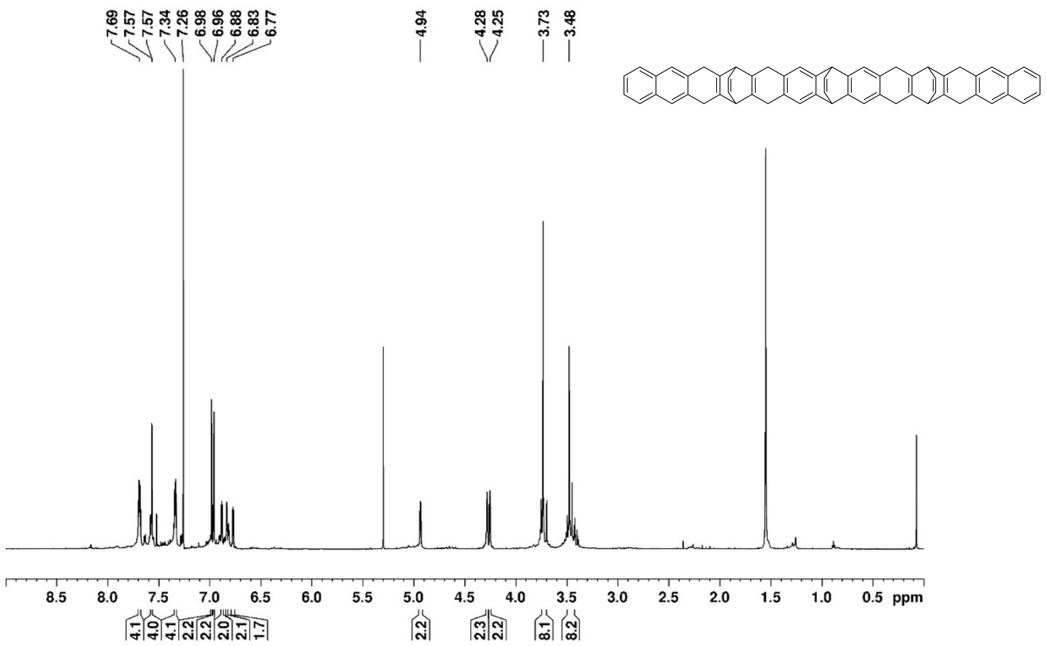

^{1}H NMR spectrum (700 MHz) of **4** in $CDCl_3$. The signal at 5.30 ppm is attributed to residues of CH_2Cl_2.

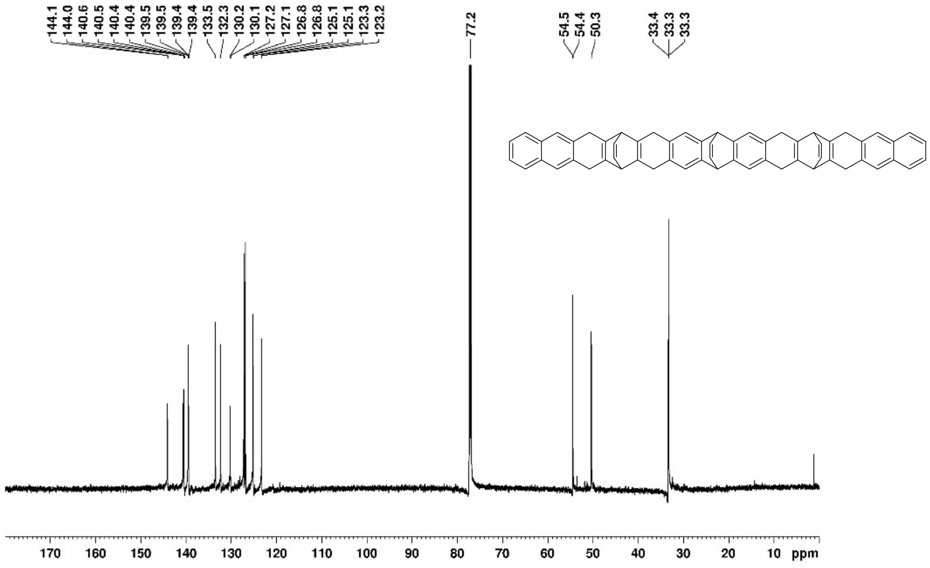

$^{13}C\{^{1}H\}$ NMR spectrum (176 MHz) of **4** in $CDCl_3$.

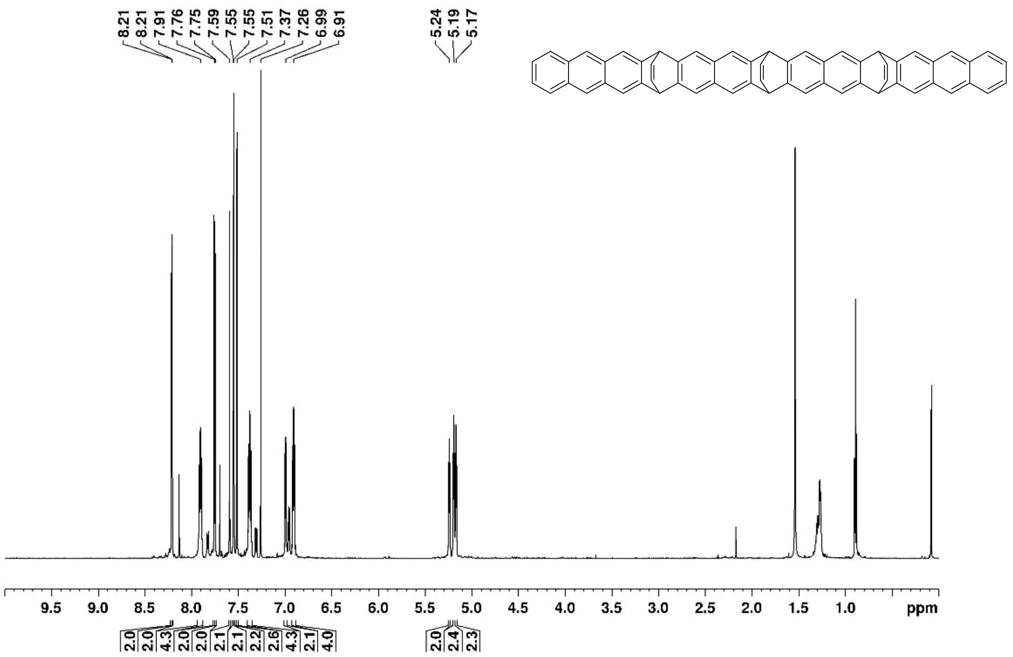

^{1}H NMR spectrum (600 MHz) of **1** in $CDCl_3$.

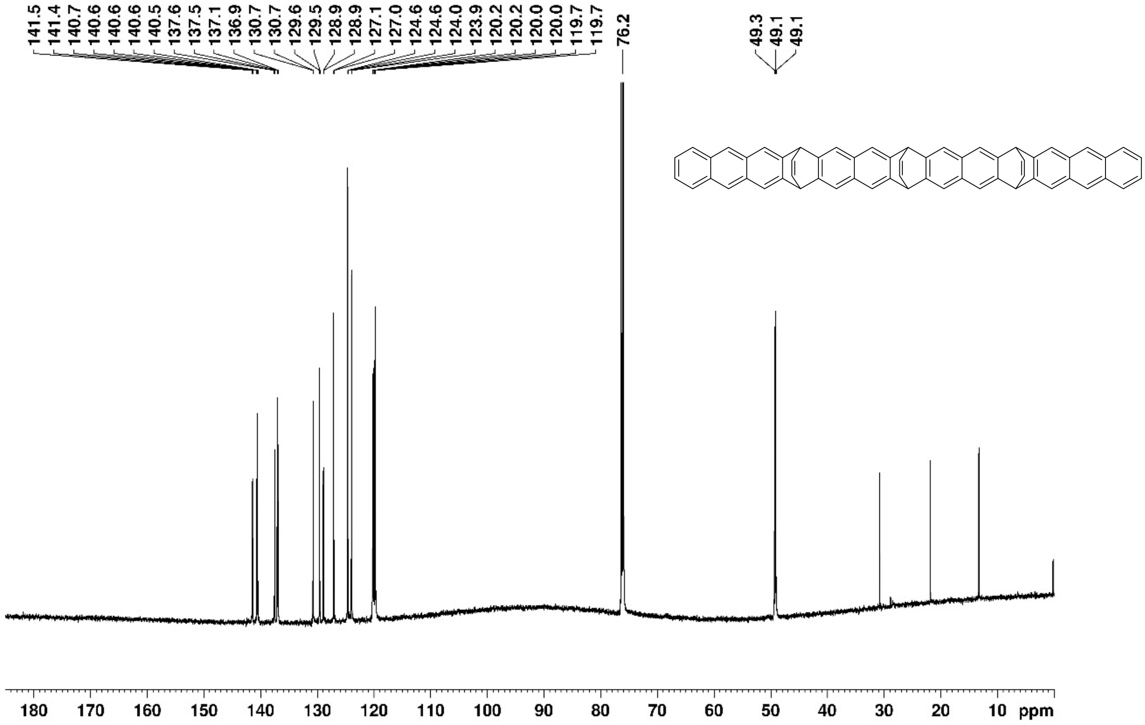

^{13}C{^{1}H} NMR spectrum (150 MHz) of **1** in $CDCl_3$. The signals between 10 and 30 ppm are attributed to residues of *n*-hexane.

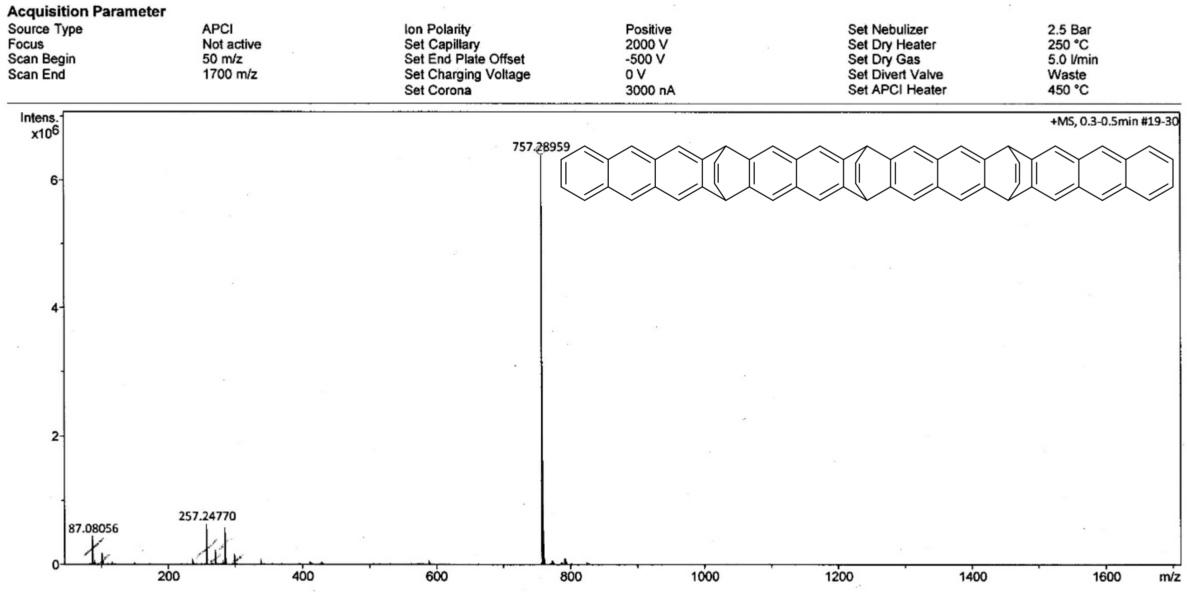

APCI mass spectrum (high resolution, full spectrum) of **1**.

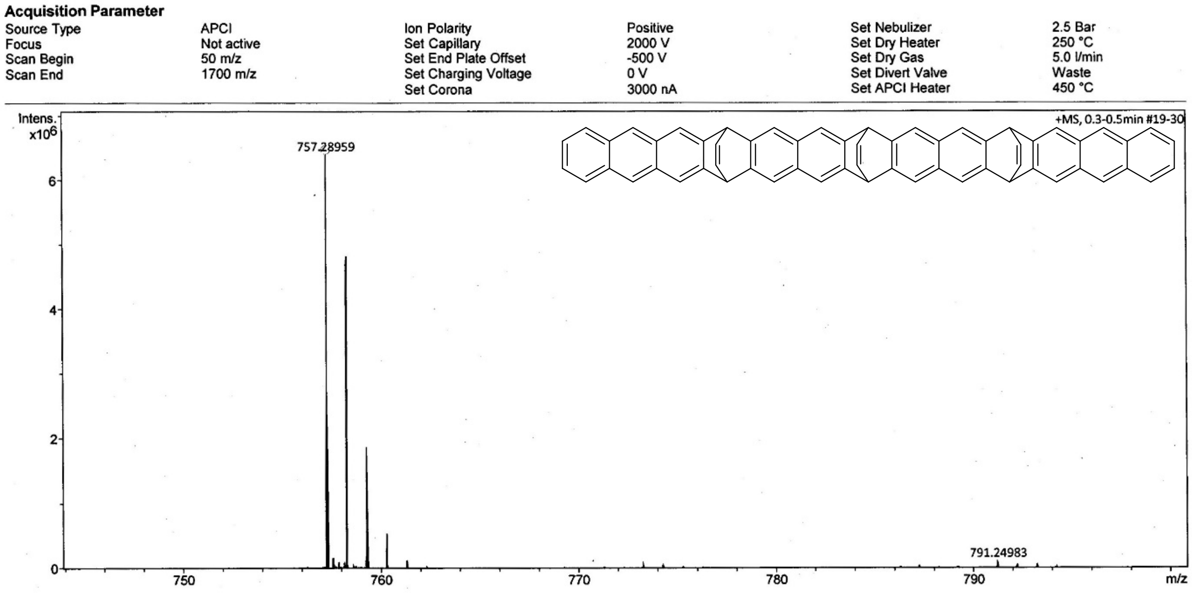

APCI mass spectrum (high resolution, magnified section) of **1**.

3. STM images of the Au(111) surface with different coverage of precursor molecule

At low coverage, the terraces are covered with smaller fragments due to the decomposition of the precursor molecules, where single intact molecules at the step edge can be found. At higher coverage, molecular island formed by edge-on adsorbed molecules are present.

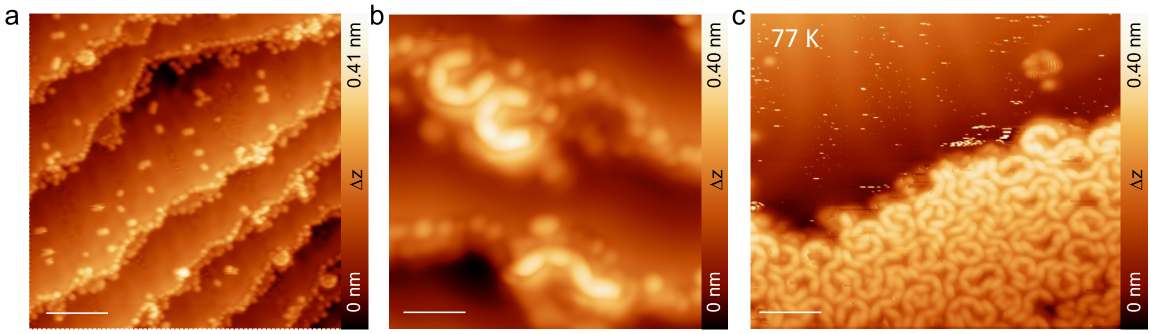

Figure S1. Au(111) surface after precursor deposition of different molecular coverage. (a) Large-scale STM image of the surface at low coverage and (b) zoom-in image of monomer precursors at the step edge. (c) Molecular island formed by intact precursor. Scale bars: (a) 7 nm; (b) 1.6 nm; (c) 3 nm. Scanning parameters: (a, b) $V_s = 0.15$ V, $I_t = 30$ pA; (c) $V_s = 1$ V, $I_t = 20$ pA.

4. *M*-shaped precursor isomer on Au(111)

Although theory demonstrates basically the same relative energies in the gas phase and adsorption energies (Table S1) compared to /111/ (*C*-shape) and $/11\bar{1}/$ (*S*-shape), the $/1\bar{1}1/$ (*M*-shaped) molecule has an extremely low proportion of molecules on the surface; only several specimens were found during the measurement. Figure S2 shows an *M*-shaped monomer at the step edge, surrounded by fragments.

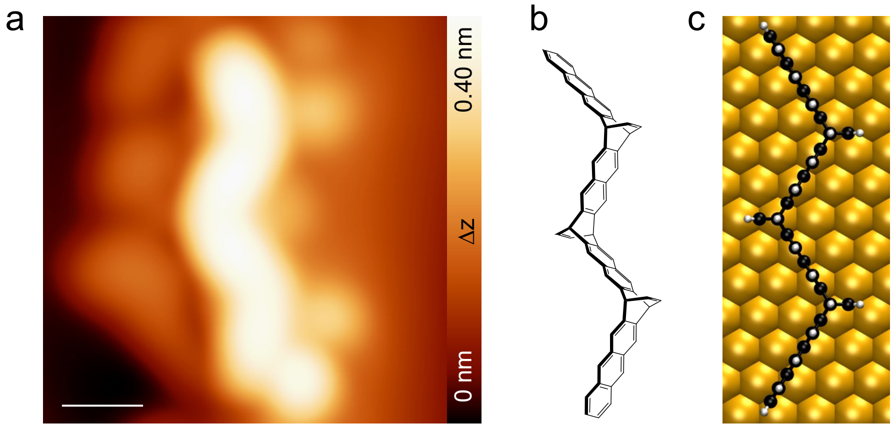

Figure S2. *M*-shaped precursor isomer on Au(111). (a) STM image of the *M*-shaped precursor molecule, (b) its chemical structure and (c) its DFT-optimized structure on the Au(111) surface. Scale bar: (a) 0.7 nm. Scanning parameters: (a) $V_s = 0.15$ V, $I_t = 50$ pA.

5. Adsorption energy and decomposition of edge-on *C*-shaped /111/, *M*-shaped /$1\bar{1}1$/ and *S*-shaped /$11\bar{1}$/ precursors

To not only get information about the magnitude of the adsorption energy, but also about the character of the bond, it can be decomposed into different energy terms. The adsorption energy E_{ads} is defined as the difference between the energy of the optimized adsorbate-surface complex $E_{\mathrm{asc}}^{\mathrm{opt}}$ and the energy of the optimized molecule $E_{\mathrm{mol}}^{\mathrm{opt}}$ and the optimized surface $E_{\mathrm{surf}}^{\mathrm{opt}}$. Additionally, it can be decomposed into a preparation energy E_{prep} and an interaction energy E_{int}.

$$E_{\mathrm{ads}} = E_{\mathrm{asc}}^{\mathrm{opt}} - \left(E_{\mathrm{mol}}^{\mathrm{opt}} + E_{\mathrm{surf}}^{\mathrm{opt}}\right) = E_{\mathrm{prep}} + E_{\mathrm{int}}$$

The preparation energy gives an idea of how much the molecule and the surface are deformed during the formation of the bond. It is defined as the energy difference between the independent fragments frozen in the adsorbate-surface complex geometry (adsorbate molecule $E_{\mathrm{mol}}^{\mathrm{frz}}$ and surface $E_{\mathrm{surf}}^{\mathrm{frz}}$) and the independently optimized fragments. Therefore, the preparation energy can be further divided into a molecule preparation energy $E_{\mathrm{prep}}(\mathrm{mol})$ and a surface preparation energy $E_{\mathrm{prep}}(\mathrm{surf})$.

$$E_{\mathrm{prep}} = \left(E_{\mathrm{mol}}^{\mathrm{frz}} - E_{\mathrm{mol}}^{\mathrm{opt}}\right) + \left(E_{\mathrm{surf}}^{\mathrm{frz}} - E_{\mathrm{surf}}^{\mathrm{opt}}\right) = E_{\mathrm{prep}}(\mathrm{mol}) + E_{\mathrm{prep}}(\mathrm{surf})$$

The interaction energy is then the bond energy between the frozen fragments and defined as the difference between the energy of the optimized adsorbate-surface complex $E_{\mathrm{asc}}^{\mathrm{opt}}$ and the energy of the frozen adsorbate molecule $E_{\mathrm{mol}}^{\mathrm{frz}}$ and the frozen surface $E_{\mathrm{surf}}^{\mathrm{frz}}$.. However, because the additive DFT-D3(BJ) dispersion correction was used, the interaction energy can be further decomposed into a dispersion interaction energy $E_{\mathrm{int}}(\mathrm{disp})$ and an electronic interaction energy $E_{\mathrm{int}}(\mathrm{elec})$

$$E_{\mathrm{int}} = E_{\mathrm{asc}}^{\mathrm{opt}} - \left(E_{\mathrm{mol}}^{\mathrm{frz}} + E_{\mathrm{surf}}^{\mathrm{frz}}\right) = E_{\mathrm{int}}(\mathrm{disp}) + E_{\mathrm{int}}(\mathrm{elec})$$

The dispersion interaction is analogously to the interaction energy the difference between the DFT-D3(BJ) energy of the adsorbate-surface complex $DE_{\mathrm{asc}}^{\mathrm{opt}}$ and the frozen fragments

(adsorbate molecule $DE_{\text{mol}}^{\text{frz}}$ and surface $DE_{\text{surf}}^{\text{frz}}$).

$$E_{\text{int}}(\text{disp}) = DE_{\text{asc}}^{\text{opt}} - \left(DE_{\text{mol}}^{\text{frz}} + DE_{\text{surf}}^{\text{frz}}\right)$$

On the other hand, the electronic interaction is analogously the difference between the Kohn-Sham energy of the adsorbate-surface complex $KS_{\text{asc}}^{\text{opt}}$ and the frozen fragments (adsorbate molecule $KS_{\text{mol}}^{\text{frz}}$ and surface $KS_{\text{surf}}^{\text{frz}}$).

$$E_{\text{int}}(\text{elec}) = KS_{\text{asc}}^{\text{opt}} - \left(KS_{\text{mol}}^{\text{frz}} + KS_{\text{surf}}^{\text{frz}}\right)$$

In the end, the adsorption energy can be decomposed the following way:

$$E_{\text{ads}} = \underbrace{E_{\text{prep}}(\text{mol}) + E_{\text{prep}}(\text{surf})}_{E_{\text{prep}}} + \underbrace{E_{\text{int}}(\text{disp}) + E_{\text{int}}(\text{elec})}_{E_{\text{int}}}$$

Applying this decomposition to the adsorption of the edge-on precursors, it is noticeable that the adsorption energy is dominated by the dispersion interaction with a repulsive (positive) electronic interaction. This demonstrates, that all edge-on precursors are physisorbed on the Au(111) surface.

Furthermore, there are no significant differences in the adsorption energy or the energy terms for the three different stereoisomers (Table S1). Also, the relative energies of the gas phase molecules are below 1 kJ mol^{-1} (Table S1). Therefore, the observed difference in occurrence with the *M*-shaped /1$\bar{1}$1/ isomer being much less abundant, cannot be explained by those results.

Table S1. Adsorption energies and energy decomposition of edge-on *C*-shaped /111/, M-shaped /1$\bar{1}$1/ and S-shaped /11$\bar{1}$/ precursors. Energies are given in kJ mol^{-1}.

	C-shaped	*M*-shaped	*S*-shaped
E_{rel}(gas)	0.5	0.0	0.3
E_{ads}	-306	-305	-306
E_{int}	-309	-308	-309
E_{int}(disp)	-341	-342	-342
E_{int}(elec)	32	34	34
E_{prep}	3	3	3
E_{prep}(mol)	1	2	1
E_{prep}(surf)	2	1	1

6. High-resolution images of the edge-on molecules

In the constant-height mode BR-STM image for a *C*-shaped molecule (Figure S3b), the two hydrogen atoms at the center of each segment show brighter feature (white arrows) due to their slightly shorter distance (~0.1 Å in DFT), as shown in Figure S3b. This observation also agrees with DFT calculations, where the two central hydrogen atoms are slightly closer together by 0.1 Å than the hydrogen atoms adjacent to the etheno groups, as shown in Figure S3c.

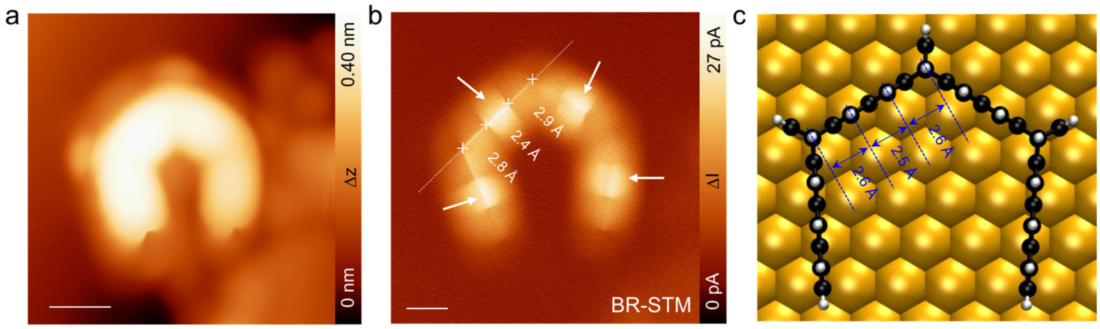

Figure S3. **High-resolution STM measurement of a *C*-shape molecule**. (a) STM, (b) BR-STM and (c) DFT-optimized structure of a *C*-shape molecule on the Au(111) surface. Scale bars: (a, b) 0.7 nm. Scanning parameters: (a) $V_s = 0.15$ V, $I_t = 30$ pA; (b) $V_s = 2$ mV.

The etheno-bridges show negligible features in constant-height mode BR-STM measurements due to their slightly lower height compared to the top edge hydrogen atoms (~1.1 Å in DFT, see Figure 1g in main text). This is confirmed by constant-height mode nc-AFM measurements and nc-AFM simulations (Figure S4b and S4c). In contrast, in constant-current mode nc-AFM measurements, the etheno-bridges as well as the two hydrogen atoms at the two ends (all in light orange color in Figure S4d) can be identified (Figure S4f).

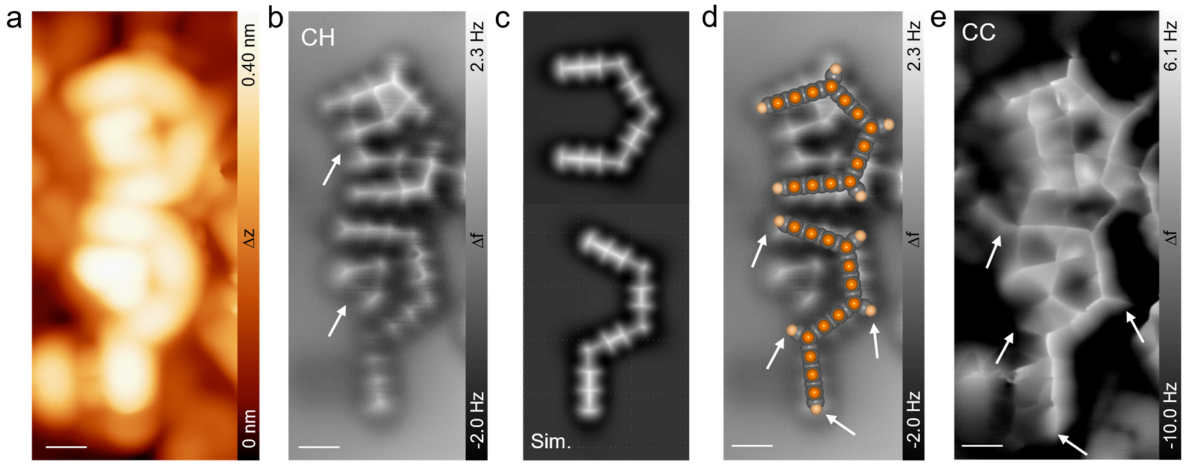

Figure S4. Structural characterization of the edge-on stereoisomers. (a) Constant-current STM image and (b) constant-height nc-AFM image of two adjacent *C*-shaped and *S*-shaped molecules. Note that there are also fragments inside the *C*-shaped region (white arrows in (b)). (c) Constant-height nc-AFM simulation using the probe particle model. (d) The same image as in (b) with overlaid molecules. (e) Constant-current nc-AFM image of the same molecules as shown in (b). White arrows in (d) and (e) highlight the lowered hydrogen atoms of the etheno-bridges and of the two ends. Scale bars: (a-e) 0.5 nm. Scanning parameters: (a) $V_s = 0.12$ V, $I_t = 30$ pA; (b) $V_s = 2$ mV; (e) $V_s = 6$ mV, $I_t = 5$ pA.

7. Conformational change during isolation

The short acene fragments and the step edge provide additional stabilization to the edge-on molecules. This can be further confirmed from its isolation process during manipulation. Figure S5 below shows the slight change of the edge-on /111/ precursor isomer when being moved from a step edge to the terrace. As a result, the distance between the two ends gets measurably smaller by 2.2 Å. Comparing the distance between the two ends of the /111/ precursor isomer measured by STM with DFT calculations, the distance is found to be overestimated of ~12 Å. However, the bending of the arms of the /111/ precursor isomer appears to have a flat potential energy surface, so another local minimum (employing the force-based convergence criteria of 10^{-2} eV $Å^{-1} \approx 1$ kJ mol^{-1} $Å^{-1}$) could be found with a lowered distance of ~9 Å, which is only 2 kJ mol^{-1} higher in energy. Such a flat potential energy surface indicates that the surface, step edges, or other fragments on the surface could easily lead to the observed deformation.

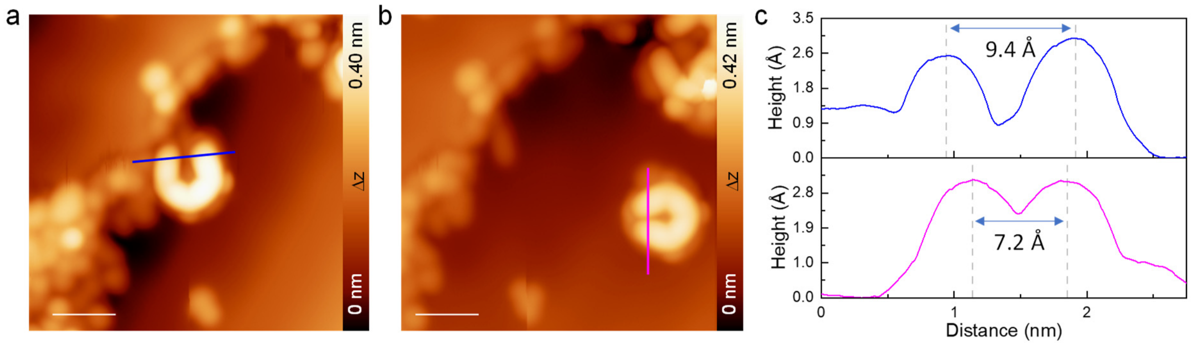

Figure S5. Isolation of a *C*-shaped molecule. (a, b) Two STM images of a *C*-shaped /111/ precursor isomer before and after its isolation from a step edge. (c) Measured distance along the lines in (a) and (b). Scale bars: (a, b) 1.5 nm; Scanning parameters: (a, b) $V_s = 0.15$ V, $I_t = 15$ pA.

8. Conversion of the /111/ isomer from its edge-on *C*-shape conformation to its stretched-out linear conformation

To induce the conformational conversion (stretching out) of a /111/ isomer from the *C*-shaped edge-on conformation to the stretched-out conformation, we first removed the surrounding fragments (Figure S6a-S6c) to ensure sufficient lateral space for the structural relaxation. The molecule then was rotated to contact the step edge at one end (Figure S6d). After that, the molecule was partially transferred to the upper step edge (Figure S6e). Further adjustments of the adsorption geometry led to the conformational change of the *C*-shaped molecule, resulting in the flat /111/ conformation (Figure S6f).

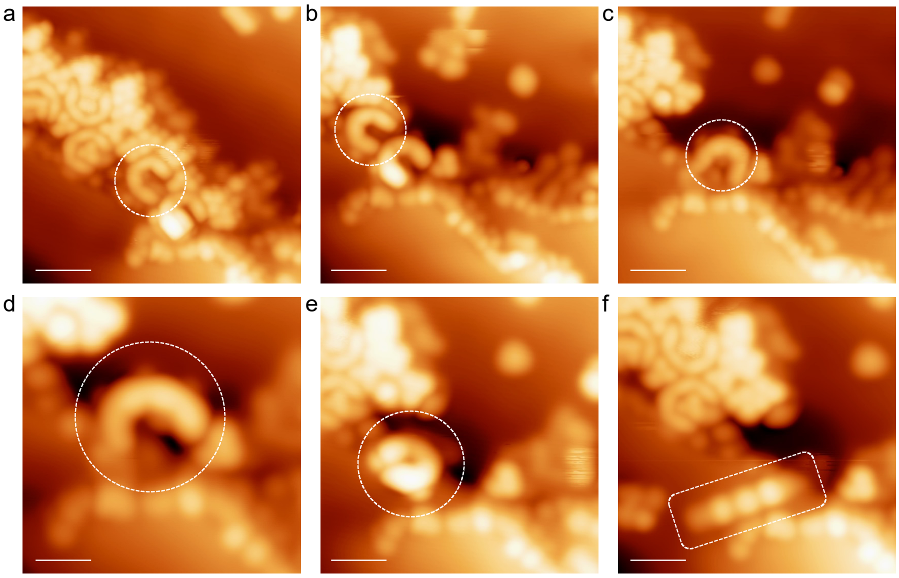

Figure S6. Tip-manipulation-induced transformation of the /111/ isomer from the edge-on *C*-shape to the stretched-out linear /111/ conformation. (a-e) Step-by-step tip manipulation of an intact precursor molecule, as indicated by a white dashed circle and (f) dashed square. Scale bars: (a-c) 2 nm; (d) 1.2 nm; (e, f) 1.6 nm. Scanning parameters: (a-c, e) V_s = 0.1 V, I_t = 40 pA; (d) V_s = 0.1 V, I_t = 30 pA; (f) V_s = 50 mV, I_t = 50 pA.

9. Stretched-out linear /111/ molecules upon deposition

After the deposition, most precursor molecules with the bridges on one side adopt the edge-on *C*-shaped conformation. Very rarely, we observed the stretched-out linear conformation of these molecules, as shown in Figure S7.

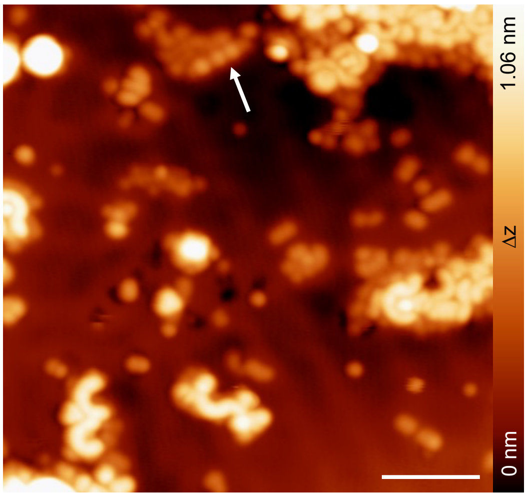

Figure S7. Stretched-out linear /111/ molecules on the surface. Linear /111/ isomer (white arrow) near some fragments. Scale bar: 5 nm. Scanning parameters: V_s = 0.15 V, I_t = 20 pA.

10. Reaction barrier estimate for edge-on *C*-shaped to stretched-out linear transition of the /111/ precursor

Since the stretched-out linear conformation of the /111/ precursor was much more rarely observed than the edge-on *C*-shaped conformation, which the molecule also has in the gas phase, we expected that the conversion from the edge-on to the stretched-out conformation has an energy barrier. To estimate this barrier, we performed NEB calculation with the frozen slab approximation. However, the frozen slab leads to a slightly less stabilizing adsorption energy because of the hindered relaxation of the surface atoms. For the edge-on *C*-shaped conformation, this error is 5 kJ mol^{-1}, while for the stretched-out linear conformation, it is 16 kJ mol^{-1}. This leads to an underestimation of the reaction energy by 11 kJ mol^{-1} (compare Figure S8 to ΔE_{ads} in Tab. S2). Still, the energy barrier should be less affected, since it is dominated by dispersion energy, which is also the case for the starting structure of the *C*-shaped conformation, to which it is determined in relative energies.

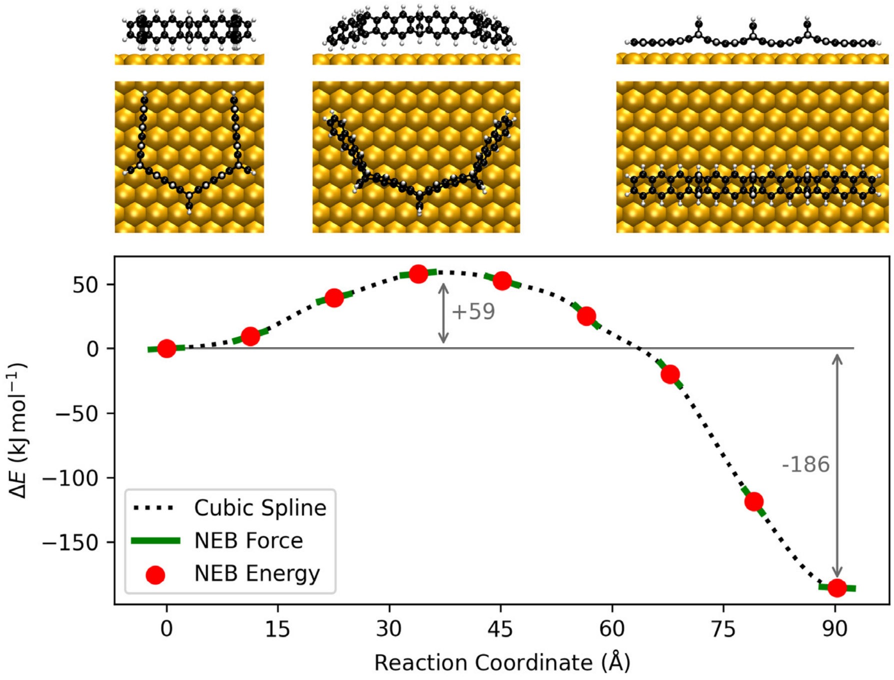

Figure S8. NEB minimum energy path for the edge-on *C*-shaped to stretched-out linear transition of the /111/ isomer. The left structure corresponds to the starting structure of the edge-on *C*-shaped conformation, the right structure to the end structure of the stretched-out linear conformation, and the middle structure to the 3rd NEB image, which is closest to the estimated maximum of the interpolation.

11. Annealing of substrate to 490 K and generation of Au incorporated 13ac by tip manipulation

To evaluate the possibility of a thermally induced on-surface C-C bond cleavage and removal of the etheno groups, we annealed the as-deposited sample up to 490 K. Instead of eliminating the etheno groups, the high mobility of the molecule leads to the decoration of step edges, which hinders further characterization. We did not see an effective cleavage of etheno groups induced by thermal annealing.

A sequential tip-induced cleavage of the etheno groups of an acene precursor at the step edge only produced an acene-like structure with higher contrast in the center, which we assign to the Au atoms incorporated **13ac** due to the readily available Au atoms from the step edges.

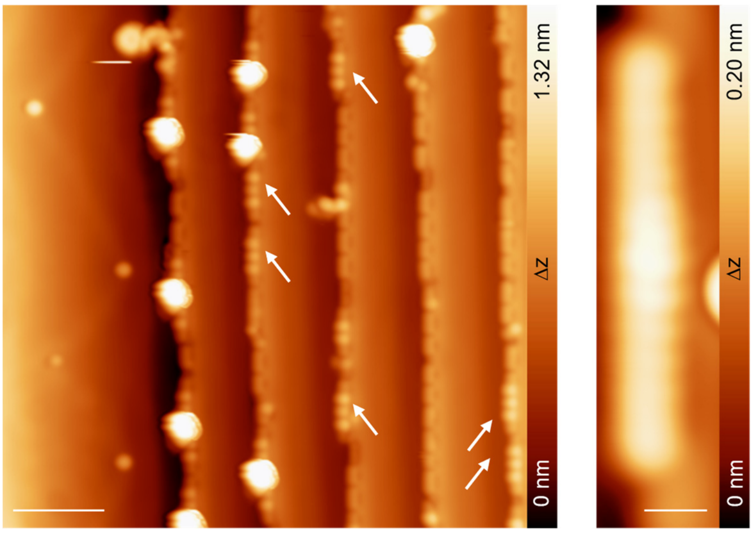

Figure S9. Precursor molecules decorating step edges after annealing. (a) STM image taken after deposition of the precursor followed by annealing the sample to 490 K. The white arrows mark the stretched-out linear intact precurSsor molecules. (b) STM image of an intermediate (presumably Au atoms incorporated **13ac**) attached to the step edge after the removal of the three etheno groups. Scale bars: (a) 6 nm; (b) 0.2 nm; Scanning parameters: (a-b) V_s = 0.15 V, I_t = 20 pA.

12. Adsorption energy and decomposition of edge-on *C*-shaped and stretched-out linear /111/ precursors

As already seen in Figure S8, the edge-on *C*-shaped to stretched-out linear transition is an exothermic reaction. The decomposition of the adsorption energy (see Section 5 for calculation details) reveals the reason for that (Table S2). Although there is increase in the total preparation energy (ΔE_{prep} = +144 kJ mol^{-1}) dominated by the deformation of the stretched-out molecule (ΔE_{prep}(mol) = +136 kJ mol^{-1}), it is overcompensated by a gain in interaction energy (ΔE_{int} = -341 kJ mol^{-1}) dominated by the dispersion interaction (ΔE_{int}(disp) = -330 kJ mol^{-1}), leading to an overall energy gain of ΔE_{ads} = -197 kJ mol^{-1}.

Table S2. Adsorption energies and decomposition for the edge-on *C*-shaped and stretched-out linear /111/ precursor, as well as the difference between them (Δ). Energies are given in kJ mol^{-1}.

	edge-on *C*-shaped	stretched-out linear	Δ
E_{ads}	-306	-503	-197
E_{int}	-309	-650	-341
E_{int}(disp)	-341	-671	-330
E_{int}(elec)	32	21	-10
E_{prep}	3	147	144
E_{prep}(mol)	1	137	136
E_{prep}(surf)	2	10	8

13. Reaction energies for cleavage of etheno-bridges

Starting from the stretched-out linear /111/ precursor, there are two possibilities of cleaving an etheno-bridge. An outer bridge could be cleaved, resulting in the /110/ intermediate, or the inner bridge could be cleaved, resulting in the /101/ intermediate. Starting from the /110/ structure, again the outer bridge or the inner bridge could be cleaved, resulting in the /010/ or the /100/ intermediate, respectively. Starting from the /101/ intermediate, only an outer bridge could be cleaved, resulting in the /100/ intermediate. Finally, cleavage of the last etheno-bridge from the /010/ or the /100/ intermediate leads to the formation of the title molecule **13ac**.

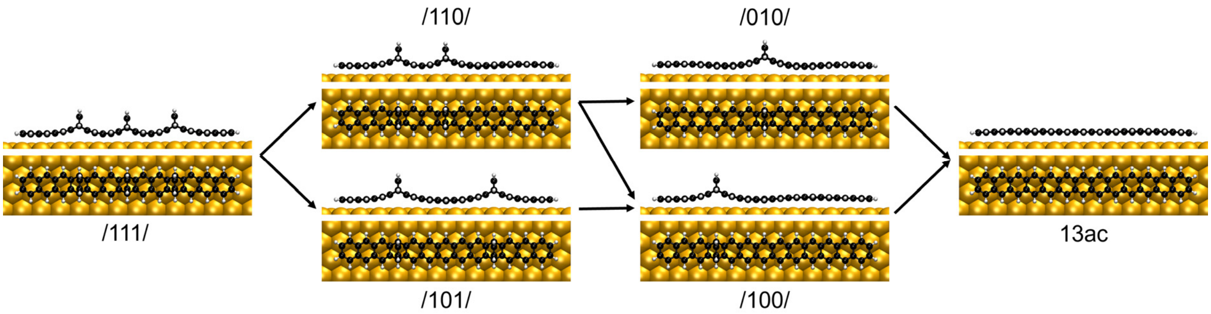

Figure S10. Optimized DFT structures of the precursor, intermediates, and final 13ac and possible reaction pathways between them.

The reaction energy for the cleavage of an etheno-bridge was calculated in such a way that the cleaved acetylene is always in the gas phase, since in experiment, after the voltage pulse, it has a momentum away from the surface towards the vacuum. The cleavage of an etheno-bridge is always endothermic (Table S3). Interestingly, it is nearly independent of which bridge is cleaved. For a hypothetical gas phase reaction, the reaction energy (ΔE_{gas}) is around 215 kJ mol^{-1}, while for the on-surface reaction (ΔE_{ads}) it is around 165 kJ mol^{-1}. The difference between them ($\Delta\Delta E$) of around 50 kJ mol^{-1} can be explained with an increase in adsorption energy, as also can be seen in Table S4.

Table S3. Reaction energies for cleavage of etheno-bridges for hypothetical gas phase (ΔE_{gas}) and on-surface reactions (ΔE_{ads}) and difference between them ($\Delta\Delta E$). Energies are given in kJ mol^{-1}.

	ΔE_{gas}	ΔE_{ads}	$\Delta\Delta E$
/111/ → /101/ + C_2H_2	+204	+139	-65
/111/ → /110/ + C_2H_2	+218	+166	-52
/110/ → /100/ + C_2H_2	+228	+159	-68
/110/ → /010/ + C_2H_2	+219	+161	-58
/101/ → /100/ + C_2H_2	+242	+186	-56
/010/ → 13ac + C_2H_2	+204	+164	-40
/100/ → 13ac + C_2H_2	+195	+165	-30
/111/ → 13ac + 3 C_2H_2	+641	+491	-151

14. Adsorption height, adsorption energy and decomposition of the stretched-out linear precursor, all possible intermediates, and 13ac

The adsorption height is defined as the distance between the respective carbon atoms and the average height of the atoms in the top-most layer of the relaxed surface slab. Note that it is plotted for the average of symmetry-equivalent carbon atom pairs along the long side of the molecule to get a general idea of the bending of the molecule. In Figure S11a the deformation of the stretched-out linear /111/ precursor leads to three peaks, corresponding to the three sp^3 carbon atoms where the etheno-bridges are attached. Furthermore, the deformation of the molecule leads to a strong bending of the short acene fragments between the etheno-bridges, resulting in a lowered adsorption height compared to **13ac**. For **13ac**, it is noticeable that it has a larger adsorption height than shorter acenes (anthracene and benzene). Interestingly, there is basically no structural difference between the adsorbed **13ac** in the antiferromagnetic and the non-magnetic state.

In Figure S11b, the height profiles of the intermediates can be seen. Notably, if the acene fragment of the intermediate at the end is large enough (>**6ac**, for /110/, /100/, and /010/), it approaches the profile of **13ac**.

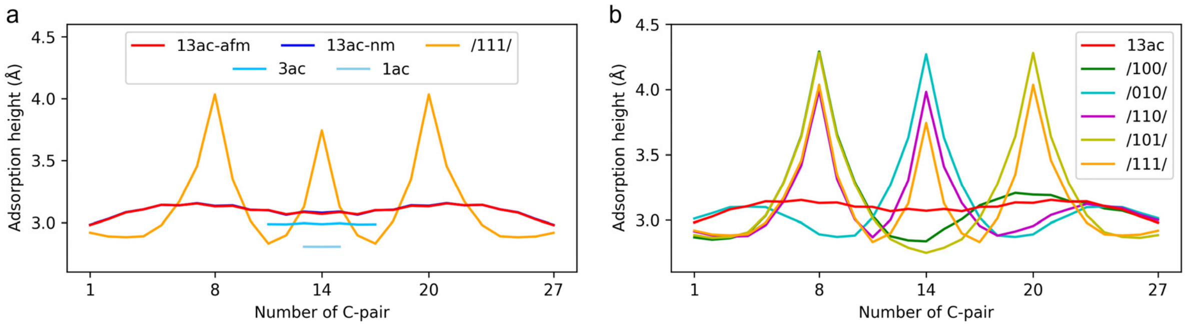

Figure S11. Adsorption height profiles of all adsorbed species on Au(111). (a) Height profile of **13ac** in antiferromagnetic (afm) and non-magnetic (nm) state, stretched-out /111/ precursor, and anthracene (**3ac**) and benzene (**1ac**) for comparison. (b) Height profile of stretched-out linear /111/ precursor, all possible intermediates, and **13ac** (afm state).

To understand how the adsorption energy changes as one moves from the stretched-out linear /111/ precursor through the intermediates to **13ac**, we can again use the energy decomposition introduced in Section 5. First, the adsorption energy roughly increases by 50 kJ mol^{-1} when moving from three etheno-bridges (/111/) to two (/110/ or /101/), from two to one (/010/ or /100/), and finally from one to zero (**13ac**). Those 50 kJ mol^{-1} are the same as observed in Table S3. However, the gain of the adsorption energy does not come from an increase in dispersion attraction, which one might expect since the precursor molecules become flatter, because this term stays rather constant around E_{int}(disp) = -670 kJ mol^{-1} (±10 kJ mol^{-1}). Instead, it stems from the release of strain introduced by the deformation, visible in the molecular preparation energy (E_{prep}). This term decreases from 137 kJ mol^{-1} for /111/ over 92 and 65 kJ mol^{-1} for /110/ and /101/, respectively, to around 40 kJ mol^{-1} for /010/ and /100/, until it is nearly negligible with 3 kJ mol^{-1} for **13ac**.

Table S4. Decomposed adsorption energies of stretched-out linear /111/ precursor, /110, /101/, /010/ and /100/ intermediates, and 13ac (afm state). Energies are given in kJ mol^{-1}.

	/111/	/110/	/101/	/010/	/100/	**13ac**
E_{ads}	-503	-555	-568	-613	-623	-653
E_{int}	-650	-655	-646	-662	-672	-661
E_{int}(disp)	-671	-675	-661	-675	-671	-683
E_{int}(elec)	21	19	15	14	0	22
E_{prep}	147	100	79	48	48	7
E_{prep}(mol)	137	92	65	40	39	3
E_{prep}(surf)	10	8	13	8	9	5

15. Reaction barrier estimation for the cleavage of an etheno-bridge from /111/ precursor to /110/ intermediate

We estimate the energy barrier for the cleavage of an etheno-bridge by performing an exemplarily NEB calculation for the reaction of /111/ to /110/ using the settings mentioned in Section 10. Again, the frozen slab approximation leads to a slightly less stabilizing adsorption energy. For the starting structure of the stretched-out linear /111/ precursor, this error has the same value as in Section 10 (+16 kJ mol^{-1}). However, for the end structure, the additional adsorption energy of the physisorbed acetylene compensates for the error introduced by the frozen slab approximation, leading to a value of -2 kJ mol^{-1}. The combination of errors in both the starting and end structures accounts for the reduced reaction energy (-18 kJ mol^{-1}) compared to the value reported in Table S3. A single point calculation of the adsorbed /110/ molecule in the end structure without the acetylene reveals the error of the frozen slab approximation with +14 kJ mol^{-1}, which is similar to the error of the /111/ starting structure. Since the energy of the reaction barrier is a relative energy term, it is expected that the error introduced by the frozen slab approximation will cancel out approximately.

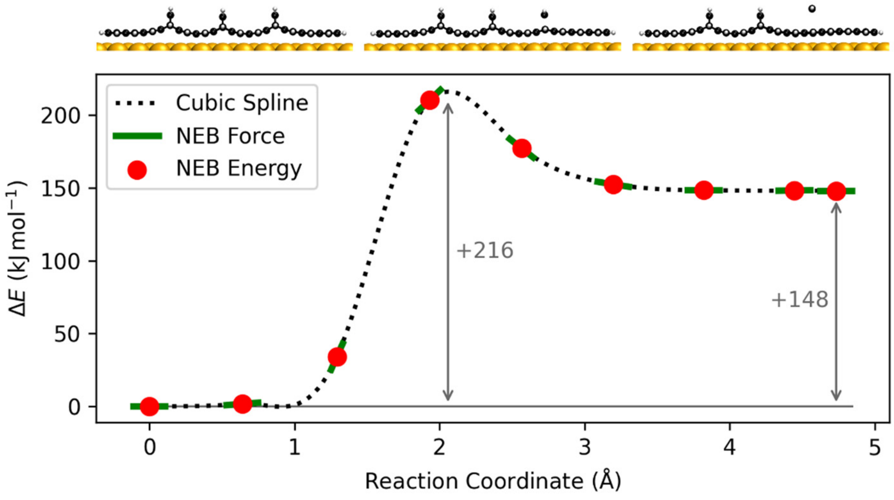

Figure S12. NEB minimum energy path for the cleavage of an etheno-bridge from /111/ to /110/. The left structure corresponds to the starting structure of the stretched-out linear /111/ precursor, the right structure to the end structure of the /110/ intermediate with an acetylene physisorbed on it, and the middle structure to the 3rd NEB image, which is closest to the estimated maximum of the interpolation.

16. Side reactions induced by the STM tip manipulation

The tip-induced removal of the etheno-bridges occasionally gives rise to various side products, which are attributed to remaining atoms of the bridges (Figure S13b) or the elimination of atoms in the annulated backbone.

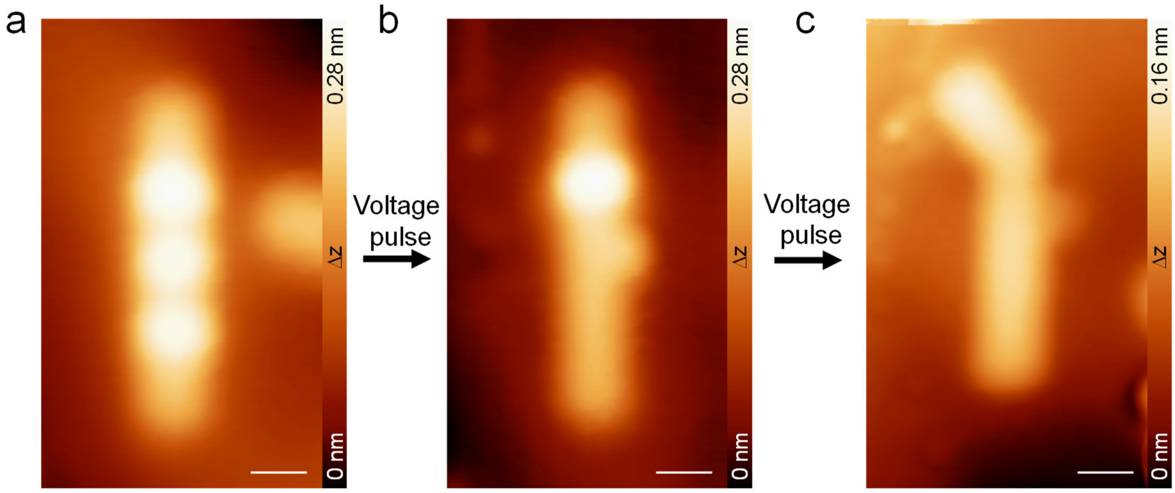

Figure S13. Side reaction during the removals of etheno-bridges. (a) Intact /111/ molecule. (b) A side product after applying a voltage pulse to the central etheno group. (c) A kinked side product after applying a pulse voltage to the third etheno group. Scale bars: (a-c) 6 Å. Scanning parameters: (a-c) V_s = -0.1 V, I_t = 30 pA.

17. Identifications of some side products after manipulation

The occasionally observed side reactions can either alter the six-membered ring underneath the etheno-bridge (Figure S14a and 14e) or the intact benzene ring (Figure S14b-14d and S14f), which might be caused by inelastic energy transfer from a non-precisely located tip and the robustness of the etheno group as well as the highly reactive intermediates.

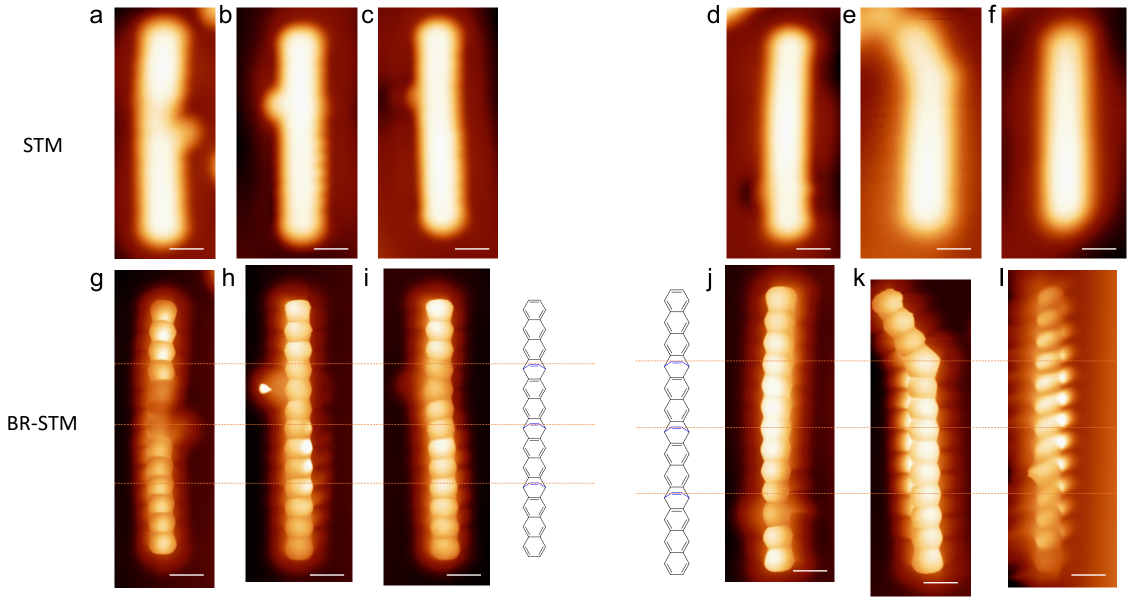

Figure S14. Observed side products after the removals of the three etheno groups. (a-f) STM images of the side products and (g-l) their corresponding BR-STM images. The dashed orange lines denote the positions of the etheno groups. Scale bars: (a, g) 3.2 Å; (b-c, h-i, l) 4 Å; (d, f) 2.4 Å; (e, j-k) 2.2 Å; Scanning parameters: (a-d) $V_s = 0.15$ V, $I_t = 15$ pA; (e) $V_s = 0.15$ V, $I_t = 6$ pA; (f) $V_s = -0.2$ V, $I_t = 30$ pA.

18. 2Au-4H-13ac complex on Au(111) surface

Figure S15b shows a straight molecule surrounded by some fragments and impurities that resembles **13ac**, while the edges show some additional features. Based on the evidence provided below, we assign this molecule as Au atom incorporated hydrogenated **13ac**. High resolution BR-STM and nc-AFM measurements identify the hydrogenated part (Figure S15d and S15e). Figure S16b and S16c show the STM image of the same molecule after removing one hydrogen atom at each sp^3 center (see also Figure S16). Now, a more homogeneous edge can be observed, while the central part remains slightly dark (bright) at negative (positive) bias. STS measurement of the resulting **2Au-13ac** complex shows two resonance peaks and the corresponding dI/dV maps also capture the electronic features from the central part (Figure S17e and S17f). Further elimination of a gold atom at the upper side of the **2Au-13ac** complex produced the pronounced lobes at the zigzag edge while it's absent the lower part at positive bias (Figure S16d). The last step removal of the Au atom produces the **13ac**, showing the symmetric features (Figure S16e).

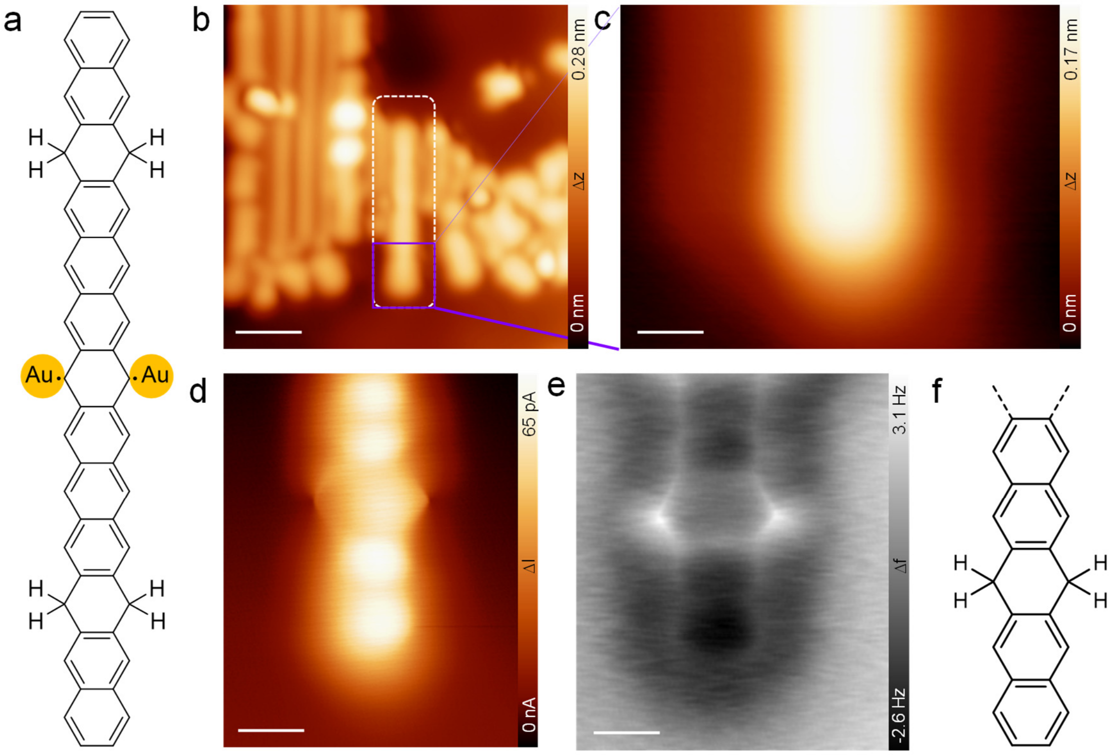

Figure S15. Au incorporated hydrogenated 13ac. (a) Proposed chemical structure of the **2Au-4H-13ac** complex and (b) experimental STM image of the **2Au-4H-13ac** complex embedded in a matrix of other molecules. (c) Zoom-in STM image of the lower hydrogenated

part. (d) BR-STM, (e) nc-AFM and (f) chemical model of the hydrogenated part. Scale bars: (b) 1.4 nm; (c) 3.2 Å; (d) 2.6 Å; (e) 3 Å; Scanning parameters: (a, b) V_s = 0.15 V, I_t = 15 pA; (d, e) V_s = 3 mV.

19. STM tip induced generation of 13ac from the Au incorporated hydrogenated 13ac

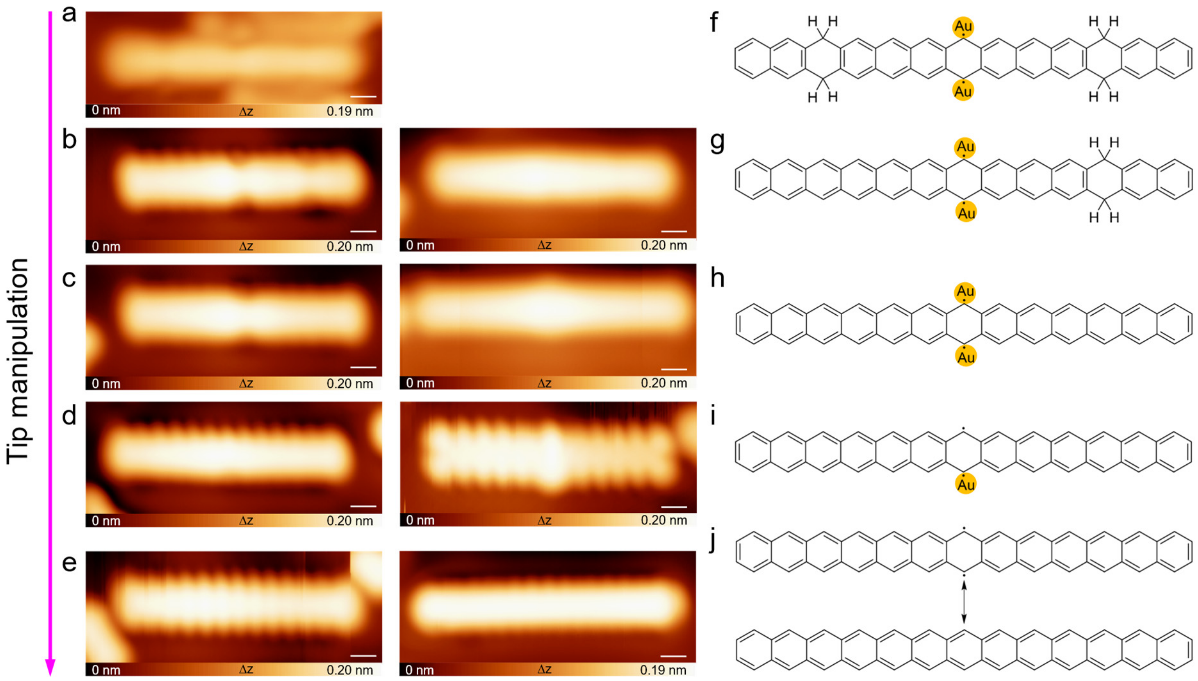

Figure S16. 13ac generated from the Au incorporated hydrogenated 13ac (2Au-4H-13ac). (a-e) STM images of the same molecule after each manipulation step. Each horizontal pair of STM images shows the same molecule imaged with different tunneling conditions. (f-j) The corresponding chemical structures of molecules shown on the left. Scale bars: (a-e) 3.2 Å; Scanning parameters: (a) V_s = 0.15 V, I_t = 15 pA; (b) (left) V_s = -0.15 V, I_t = 6 pA; (right) V_s = -0.8 V, I_t = 8 pA; (c) (left) V_s = -0.15 V, I_t = 10 pA; (right) V_s = 0.8 V, I_t = 7 pA; (d) (left) V_s = 0.08 V, I_t = 35 pA; (right) V_s = -1 V, I_t = 40 pA; (e) (left) V_s = -0.4 V, I_t = 30 pA; (right) V_s = 0.15 V, I_t = 10 pA.

20. Electronic properties of the 2Au-13ac complex

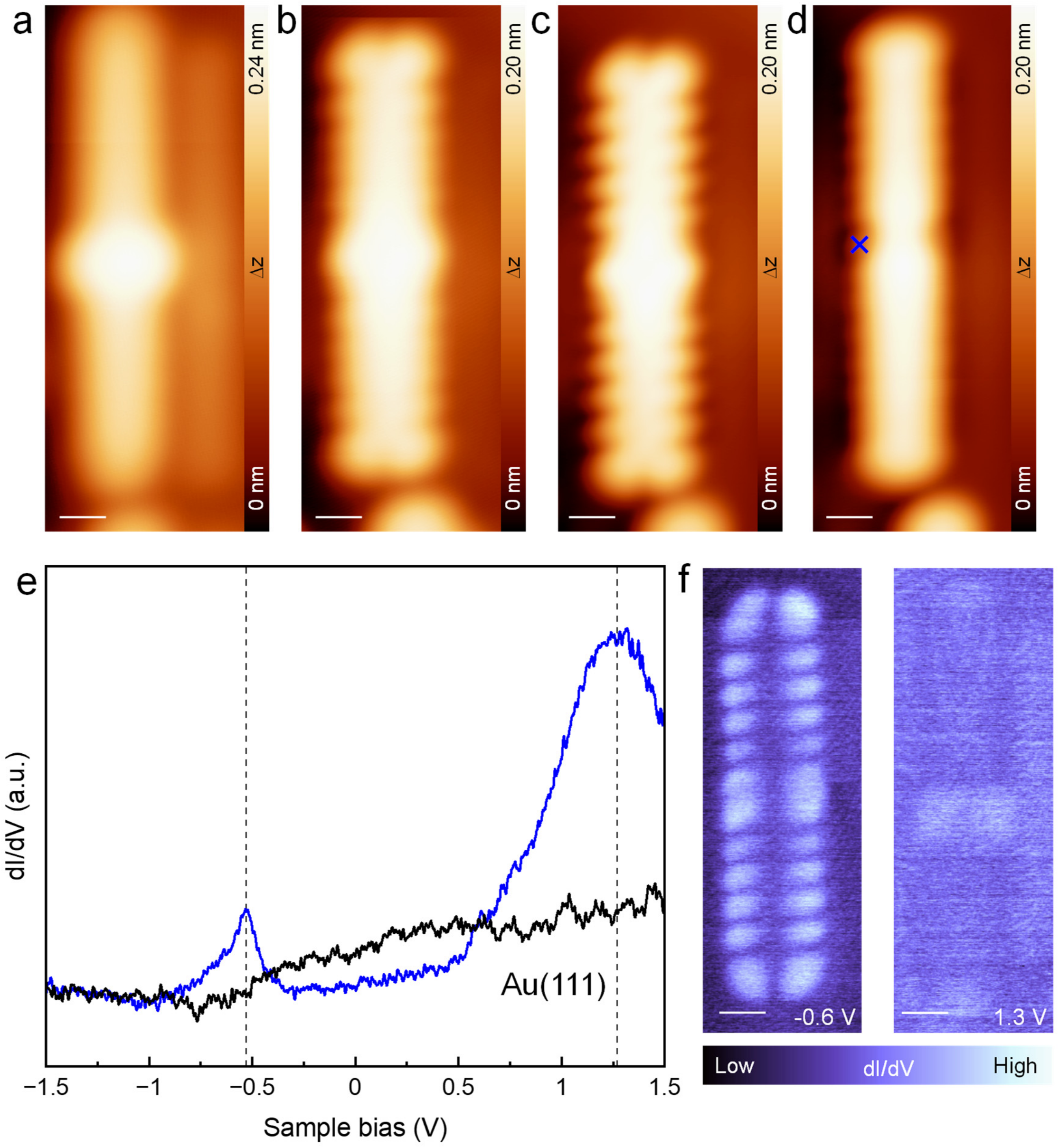

Figure S17. STS measurements on the Au-13ac complex. (a-d) Series of STM images obtained at different bias voltages. (e) STS spectrum (blue line) of the **Au-13ac** complex taken at the position marked in (d). (f) dI/dV maps taken at the bias voltages marked by the dashed lines in (e). Scale bars: (a-d, f) 3.2 Å. Scanning parameters: (a) V_s = 1.5 V, I_t = 7 pA; (b) V_s = -1.5 V, I_t = 6 pA; (c) V_s = -0.6 V, I_t = 40 pA; (d) V_s = 0.06 V, I_t = 20 pA.

21. Additional dI/dV maps of 13ac

We detected the HOMO of **13ac** around 0.35 V, which agrees well with the trend that the HOMO energy remains almost constant for acenes longer than **8ac.** The LUMO position is further confirmed by conducting an STS line map (Figure S19b) across one end of the 13ac molecule, by which a pronounced peak feature (LUMO) is again unambiguously identified. This observation can be further confirmed by comparing the amplitude of the local density of state, where the highest amplitude is observed between 0.5 V and 0.8 V.

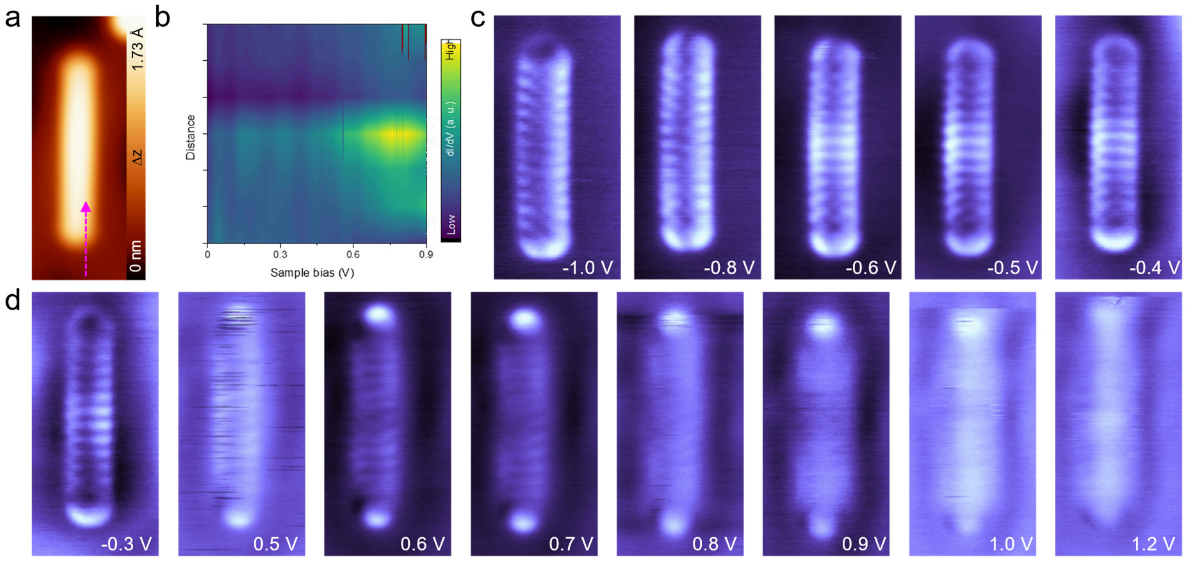

Figure S18. Additional dI/dV maps of 13ac in a wide range of bias voltages. (a) STM of **13ac**. (b) STS line map obtained at the trajectory show in a). (c) Series of dI/dV maps at different bias voltages. (d) Continuation of (c).

22. Gas phase properties of antiferromagnetic and non-magnetic acenes (7ac to 15ac)

Figure S19 shows the results for the non-magnetic (nm) and antiferromagnetic (afm) states of a series of acenes (**7ac** to **15ac**) in the gas phase. The results by Jiang and Dai[1] regarding the relative energy between the nm and afm state are reproduced using essentially the same computational settings (Figure S19a). The result of the oscillating HOMO-LUMO gap for the nm (closed-shell) state first described by Korytár *et al.*[2] is also reproduced using the same exchange-correlation functional (PBE) with a different basis set (plane waves instead of numeric atom-centered orbitals). Note that the strong oscillation of the HOMO-LUMO gap is reduced for the afm states, but there is still a slight oscillation visible.

Results by Trinquier *et al.*[3] of the spin density distribution on the outer carbon atoms are qualitatively confirmed (Figure S19c). Spin-polarized DFT with the PBE functional used here yields the first diradical character for **8ac**. On the one hand, this can be seen from the relative energy between the afm and nm state (Figure S19a). On the other hand, it can be seen in the projected magnetization (Figure S19c), where there is a jump from **7ac** to **8ac**. The first tetraradical character is observed for **14ac** in the atom-projected magnetization (Figure S19c), where two maxima occur for the first time and are maintained for **15ac**. Similarly, broken-symmetry DFT and B3LYP used by Trinquier *et al.*[3] gives the first diradical character for **7ac** and the first tetraradical character for **13ac**. However, as also shown by Trinquier *et al.*[3], the exact length at which the radical character changes depend on the amount of Hartree-Fock exchange used, which is fundamentally different between PBE (0%) and B3LYP (20%).

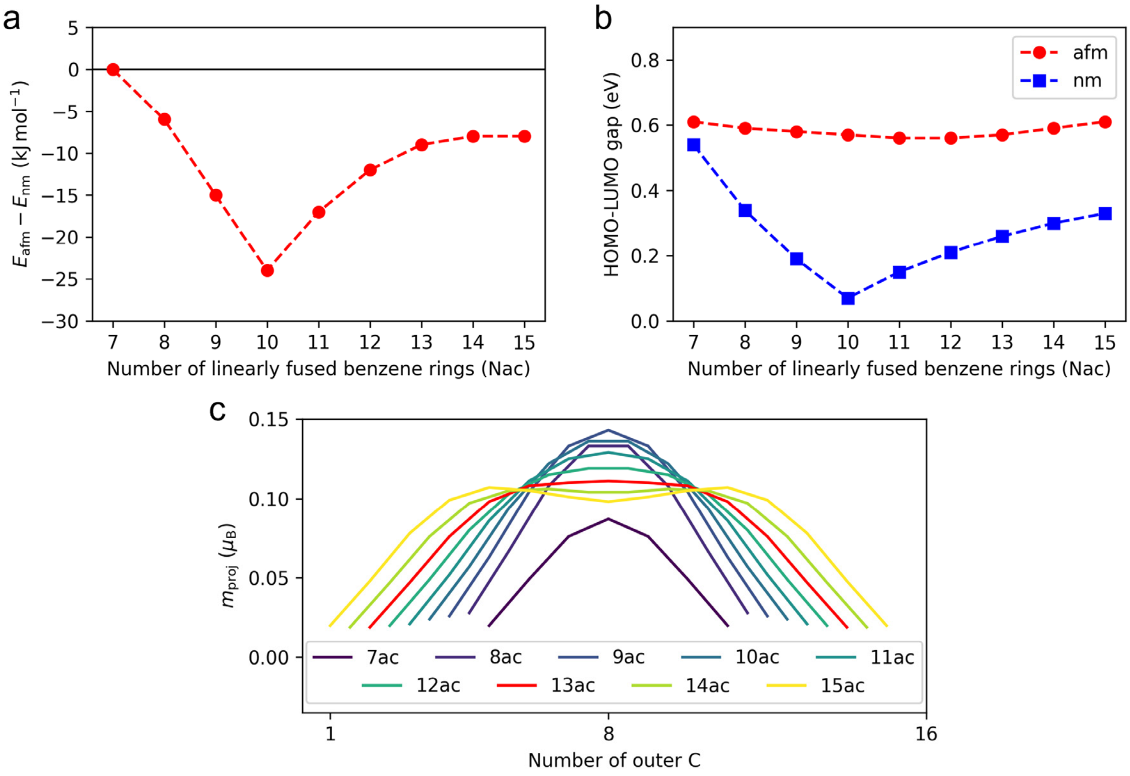

Figure S19. Gas phase properties of afm and nm acenes. (a) Relative energy between afm and nm state. As can be seen, the antiferromagnetic (afm, radical or open-shell) ground state of **13ac** is more stable by 9 kJ mol^{-1} than the non-magnetic (nm) state. (b) HOMO-LUMO gap for afm (red) and nm (blue) state. (c) Atom-projected magnetization for outer major-spin carbon atoms.

23. Relative energies and adsorption energies for antiferromagnetic and non-magnetic adsorbed 13ac

The energy difference between the afm and nm states is -9 kJ mol^{-1} in gas phase **13ac**, but only -2 kJ mol^{-1} in adsorbed **13ac**, making these states nearly isoenergetic in adsorbed **13ac**. The reason for this adsorption-induced change is revealed by the adsorption energy and its decomposition (see Section 5 for calculation details). In particular, the stabilizing adsorption energy for the afm state is lowered by 8 kJ mol^{-1}. When looking at the decomposition in Table S5, it comes exclusively from an increase in electronic repulsion. The fact that the dispersion energy does not change is consistent with the adsorption height profiles of **13ac** in the afm and nm states shown in Figure S11a, where almost no structural change is visible, but on which the DFT-D3(BJ) dispersion energy is calculated.

Table S5. Relative energies between afm and nm state of 13ac in the gas phase and adsorbed on Au(111), as well as adsorption energies and decomposition of adsorbed 13ac in afm and nm state, and difference between them (Δ). Energies are given in kJ mol^{-1}.

	**13ac**-afm	**13ac**-nm	Δ
E_{rel}(gas)	-9	0	
E_{ads}	-653	-661	+8
E_{int}	-661	-670	+9
E_{int}(disp)	-683	-683	0
E_{int}(elec)	22	12	+9
E_{prep}	7	9	-1
E_{prep}(mol)	3	4	-1
E_{prep}(surf)	5	5	0
E_{rel}(ads)	-2	0	

24. Magnetization for adsorbed 13ac in the antiferromagnetic state

The outer carbon atoms carry most of the magnetization (Figure S20b and S20c), which is in line with the view that acenes are the narrowest zig-zag edge graphene nanoribbons and thus are anticipated to possess spin-polarized edge states. Upon adsorption, the magnetization is reduced, which can be seen visually in comparison between Figure S20b and S20c. This is also observed in the atom-projected magnetization of the outer carbon atoms (Figure S20a). However, there are small differences in the magnetization of spin α and spin β on the different sides of the molecules, which comes from the different positions of the carbon atoms above the surface atoms.

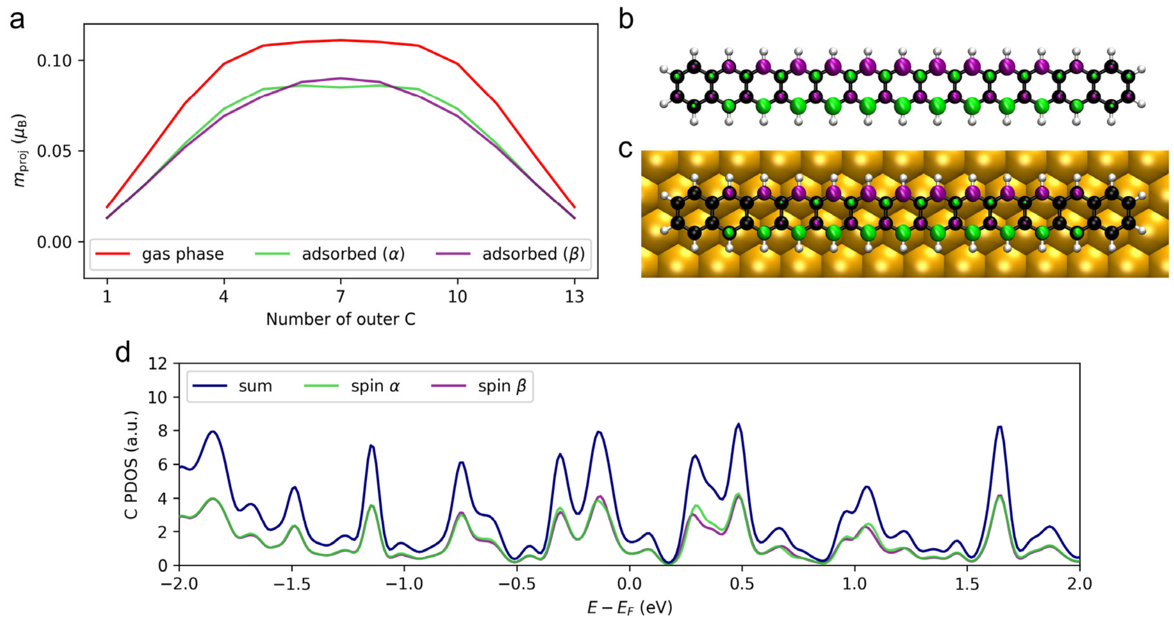

Figure S20. Magnetization for 13ac in the afm state. In all figures, spin α is shown in green and spin β in purple. (a) Atom-projected magnetization for the outer major-spin carbon atoms of gas phase (red) and adsorbed **13ac**, broken down in spin α and spin β. (b) Magnetization density (ρ_α-ρ_β) of gas phase **13ac** in afm state. (c) Magnetization density of adsorbed **13ac** in afm state with same isovalue as in (b). (d) Carbon PDOS of adsorbed **13ac** in afm state, broken down in spin α and spin β.

Although in Figure S20c, most of the magnetization density is located at the **13ac** molecule, the gold slab modeling the surface also carries magnetization. Most of the spin α magnetization present in the cell is located at Au atoms, while nearly no spin β is located there, making the

slab ferromagnetic (Table S6). This, however, is a very unlikely situation in experiment since the Au(111) surface is not known to be ferromagnetic.

Still, there are several reasons, why we assume that this will not have an artificial effect on the magnetization of the adsorbed **13ac**. First, we observe a nearly symmetric distribution of spin α and spin β magnetization in the adsorbed **13ac** (Figure 20a). Therefore, it is very unlikely that the ferromagnetism of the slab reduced the magnetization of the molecule because we would expect it to happen in an antisymmetric fashion. Second, there is no significant difference found in the carbon PDOS when breaking it down into the different spins (Figure 20d), again supporting the argument that the ferromagnetism of the slab has no influence on the magnetization of the **13ac**. Third, when normalizing the magnetization of the gold atoms, one gold atom carries only 0.003 μ_B magnetization, which is almost negligible in comparison to the 0.013 to 0.090 μ_B located at the carbon atoms of the **13ac**. Fourth, surprisingly, a ferromagnetic solution can be found for the isolated slab, which is 4 kJ mol^{-1} more stable than the non-magnetic solution and has a magnetization of 16.955 μ_B, corresponding to 0.025 μ_B per gold atom. However, the magnetization of the slab with the **13ac** adsorbed is much lower than the "true" ferromagnetic solution for the isolated slab (2 vs. 17 μ_B), meaning that a potential energy gain by magnetization of the slab is negligible. Even if this unphysical magnetization of the slab with the **13ac** adsorbed would lead to an artificial stabilization, this would strengthen the argument that the adsorbed **13ac** in afm state is nearly isoenergetic to the adsorbed **13ac** in nm state (-2 kJ mol^{-1}) and lost stabilization compared to the gas phase (-9 kJ mol^{-1}).

Table S6. Atom-projected magnetization of spin up (α) and spin down (β) for gas phase and adsorbed 13ac. Magnetization is given in μ_B

	m_{proj}(gas phase)		m_{proj}(adsorbed)	
	α	β	α	β
C	1.453	1.453	1.071	1.070
H	0.018	0.018	0.011	0.011
Au	-	-	1.973	0.022
sum	1.471	1.471	3.055	1.103
Δ	0		1.952	

25. Comparison of dI/dV maps and STM simulations

We also tried to simulate dI/dV STM maps using the Tersoff-Hamann approximation. However, a fundamental problem of closed-shell (non-magnetic) DFT for higher acenes could also be observed for the antiferromagnetic solution of spin-polarized DFT: A change in the symmetry of the HOMO and LUMO (the sum of the two isoenergetic HOSOs and LUSOs, respectively). For smaller acenes, the LUMO exhibits a mirror axis along the long molecular axis when looking perpendicular to the ring plane (the phase of the orbital at opposing carbons is the same) while the HOMO does not (the phase of the orbital at opposing carbons is not the same). Using closed-shell DFT, this suddenly changes from **11ac** on, and the HOMO now has the mirror axis while the LUMO does not (for **13ac** see Figure S21i and S21r). Up to now, this behavior has been observed in all closed-shell DFT work treating longer acenes independent of the functional used. We also tested a range of exchange-correlation functionals (PBE and PBE0 in VASP; PBE, HSE03, HSE06, B3LYP, KMLYP, mPW1K, M06-2X in ADF 2021.106) and found qualitatively similar results throughout.

The experimentally measured LUMO has characteristic lobes, one at each end (Figure S21g), which are also known for all smaller acenes.[4] Those lobes indicate that the LUMO exhibits a symmetry axis along the long axis of the molecule when looking perpendicular to the ring plane. However, those lobes show up for the STM simulation of the HOMO in the nm gas phase **13ac** (Figure S21e), which has the corresponding mirror axis (Figure S21i). This indicates that the change in symmetry based on DFT, which appeared around **11ac**, could be a computational artifact.

In contrast, the afm gas phase molecule does not show such lobes in the STM simulations, neither using the HOMO nor the LUMO (Figure S21b and S21k). In general, the STM simulation of the HOMO and LUMO look relatively similar, and the difference in symmetry can only be seen at high isovalues (Figure S21c and S21l). Looking at the isosurface of the sum of the isoenergetic HOSOs and LUSOs (Figure S21g and S21p), respectively, the difference in symmetry is more obvious and they appear relatively similar to the HOMO and LUMO of the nm state (Figure S21i and S21r). So there, the symmetry is changed as well. However, when using the adsorbed **13ac** for simulation, at least for the LUMO, the corresponding symmetry is evident (Figure S21j), although this could be an artificial effect, because it came to an artificial

splitting of the LUMO in the carbon PDOS for the Gamma-only calculation (STM simulations could not be done for a larger k-grid).

Of course, the above discussion is only valid if the one-electron picture using orbitals is a good description of the system and electron correlation effects are small. Since **13ac** exhibits static correlation effects, this is not strictly the case. Instead, in a multiconfigurational picture, also doubly excited configurations can contribute to the measured dI/dV maps.[4] Still, the dI/dV maps at negative and at positive bias voltage, respectively, should be mainly dominated by the HOMO and LUMO contribution, respectively. Therefore, the missing change of symmetry of the measured dI/dV maps could be an important indication that this theoretically predicted change in symmetry of the HOMO and LUMO is an artifact of the computational mean-field approach. Another indication is that Dyson orbitals based on multiconfigurational CASCI(12,12) calculations show the symmetries observed in experiment[5]. Nevertheless, because **13ac** is only physisorbed on the Au(111) surface without stabilizing covalent interactions, we think that our observations and conclusions to the multiradical character and HOMO-LUMO gap on the surface are valid.

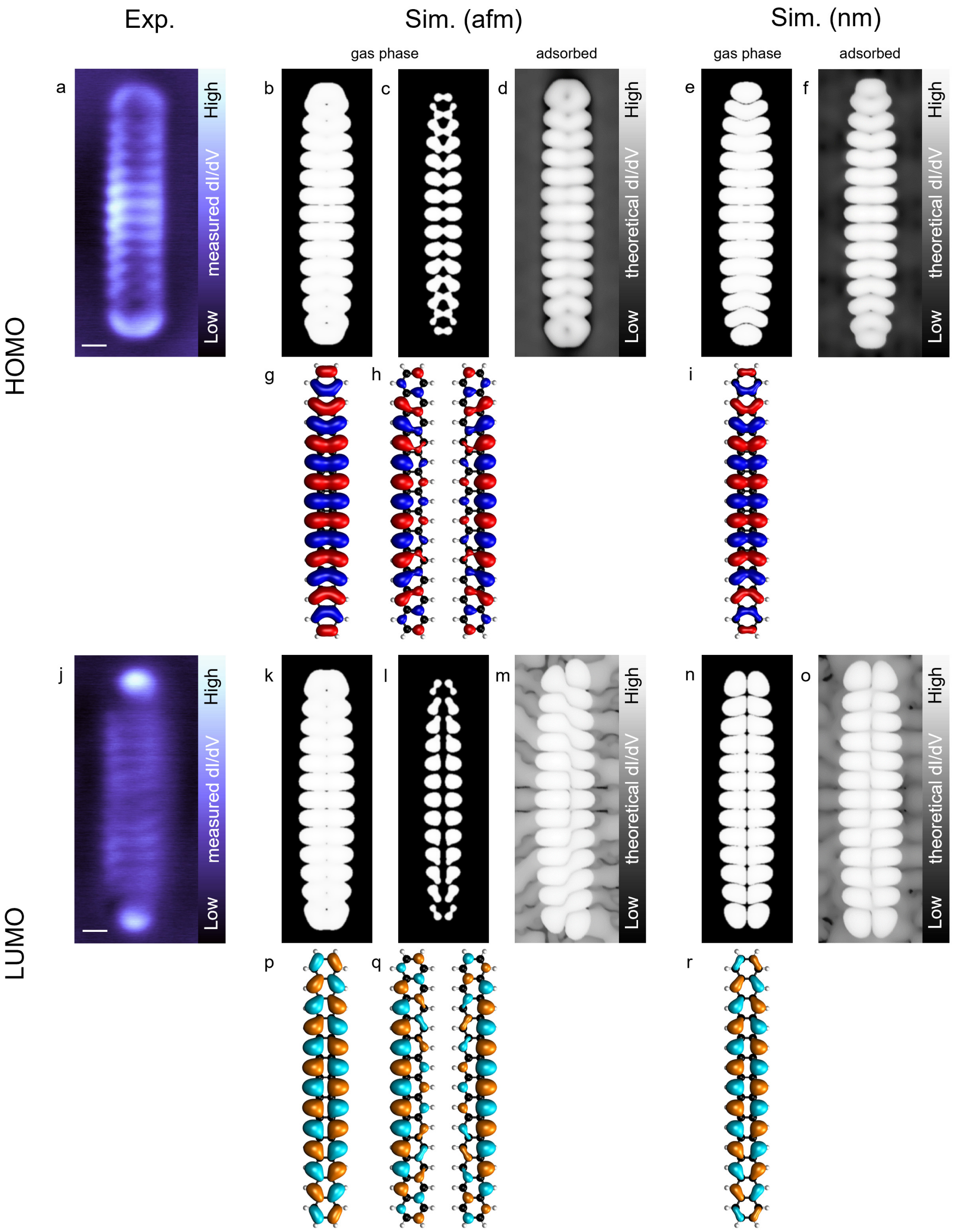

Figure S21. Measured dI/dV maps and STM simulations of 13ac using different settings. (a) dI/dV map measured near the negative resonance voltage of the STS curve (Figure 4a) . (b)-(f) STM simulation of HOMOs for the described systems. Note that (c) is the same as (b) with a larger isovalue. (g)-(i) Isosurface of highest occupied orbitals for gas phase **13ac**. Note that (g) is the sum of the two HOSOs in (h). (j) dI/dV map measured near the positive resonance voltage. (k)-(o) STM simulation of LUMOs for the described systems. Again, (l) is the same as (k) with a larger isovalue. (p)-(r) Isosurface of lowest unoccupied orbitals for gas phase **13ac**. Again, (p) is the sum of the two LUSOs in (q).

References

(1) Jiang, D.; Dai, S. Electronic Ground State of Higher Acenes. *J. Phys. Chem. A* **2008**, *112*, 332–335. 10.1021/jp0765087.

(2) Korytár, R.; Xenioti, D.; Schmitteckert, P.; Alouani, M.; Evers, F. Signature of the Dirac Cone in the Properties of Linear Oligoacenes. *Nat. Commun.* **2014**, *5*, 1–7. 10.1038/ncomms6000.

(3) Trinquier, G.; David, G.; Malrieu, J.-P. Qualitative Views on the Polyradical Character of Long Acenes. *J. Phys. Chem. A* **2018**, *122*, 6926–6933. 10.1021/acs.jpca.8b03344.

(4) Krüger, J.; Eisenhut, F.; Skidin, D.; Lehmann, T.; Ryndyk, D. A.; Cuniberti, G.; García, F.; Alonso, J. M.; Guitián, E.; Pérez, D.; Peña, D.; Trinquier, G.; Malrieu, J.-P.; Moresco, F.; Joachim, C. Electronic Resonances and Gap Stabilization of Higher Acenes on a Gold Surface. *ACS Nano* **2018**. 10.1021/acsnano.8b04046.

(5) Zuzak, R.; Kumar, M.; Stoica, O.; Soler, D.; Brabec, J.; Pernal, K.; Veis, L.; Blieck, R.; Echavarren, A.; Jelinek, P. Godlewski, S. On-Surface Synthesis and Determination of the Open-Shell Singlet Ground State of Tridecacene. *ChemRxiv*. Cambridge: Cambridge Open Engage. **2023**.10.26434/chemrxiv-2023-gkx45.
